# Vision–Floor Sensor Fusion for Non-Wearable Indoor Fall Detection Using Skeletal Motion and Distributed Force Sensing

**DOI:** 10.3390/s26216825

**Published:** 2026-10-28

**Authors:** Mifal Jacob, Utkaarsh Saha, Shabnam Sadeghi Esfahlani

**Affiliations:** School of Engineering and the Built Environment, Faculty of Science and Engineering, Anglia Ruskin University, Bishop Hall Lane, Chelmsford CM1 1SQ, UK; mj760@student.aru.ac.uk (M.J.); us211@student.aru.ac.uk (U.S.)

**Keywords:** fall detection, sensor fusion, YOLOv8, pose estimation, load cell, RGB vision, ambient assisted living, human activity recognition, ROS2, smart healthcare

## Abstract

Falls represent a critical public health concern, particularly for older adults and individuals with disabilities, often resulting in serious injuries, loss of independence, and increased mortality. Traditional fall detection systems suffer from high false-positive rates, privacy concerns, and limited real-world applicability. This study presents an improved non-wearable fall detection method integrating YOLOv8-based skeletal pose estimation with load distribution sensing through decision-level sensor fusion. The system employs YOLOv8 for real-time human posture analysis and skeletal motion-based fall recognition, addressing limitations of simple presence-based or LiDAR-only approaches. A distributed load-sensing array embedded in the sensing surface monitors relative floor-loading patterns and impact-related changes. Decision-level fusion with temporal filtering combines skeletal postural and motion information with floor-load evidence, enabling accurate fall detection with substantially fewer false positives than the vision-only configuration. Key methodologies include detailed load sensor calibration, time-domain filtering to distinguish intentional lying from falls, and fusion logic leveraging the complementary strengths of visual and floor-based load sensing. Evaluation across 160 controlled trials involving eight healthy participants achieved an F1-score of 0.935, sensitivity of 90.0%, and specificity of 97.5%. Compared with the vision-only configuration, the fused framework reduced false-positive detections by 81.8%, although sensitivity decreased from 93.8% to 90.0%. This modular ROS2-based system provides a practical, non-intrusive framework with potential for future deployment in residential and assisted-living settings, while supporting extension to broader assistive monitoring applications.

## 1. Introduction

### 1.1. Background and Motivation

Falls represent one of the leading causes of injury-related mortality and disability worldwide, particularly among older adults and individuals with impaired mobility. According to the World Health Organization, falls are the second leading cause of unintentional-injury deaths worldwide, after road traffic injuries, with adults over 60 years of age experiencing the greatest number of fatal falls [1]. Beyond immediate physical injuries, falls can result in prolonged immobilisation, loss of independence, psychological trauma, reduced quality of life, and substantial healthcare expenditure. Falls also impose a considerable economic burden on healthcare services in the United Kingdom, costing the National Health Service (NHS) more than £2.3 billion annually [2]. Delayed recognition of a fall can further increase the risk of secondary complications, including dehydration, hypothermia, pressure ulcers, rhabdomyolysis, and adverse outcomes associated with prolonged periods spent on the floor, commonly referred to as a “long lie”. Consequently, reliable and timely fall detection remains an important research challenge within ambient assisted living, intelligent healthcare, and smart-home monitoring systems.

A wide range of sensing technologies has been investigated for automatic fall detection. Wearable systems based on inertial measurement units (IMUs) can identify characteristic acceleration and angular-velocity patterns associated with falls. However, their effectiveness depends on continuous user compliance, regular battery charging, and correct device placement, which can limit their suitability for older adults and individuals with cognitive impairment [3]. Vision-based approaches provide richer contextual information through continuous observation of human activity without requiring body-worn devices. Nevertheless, conventional RGB camera systems remain sensitive to illumination variation, occlusion, camera positioning, and privacy concerns, potentially limiting their deployment in residential environments [4]. Ambient sensing approaches, including floor-vibration sensing, acoustic monitoring, and pressure-sensitive flooring, provide unobtrusive monitoring while potentially reducing visual privacy concerns. However, such methods may provide limited contextual information for distinguishing genuine falls from activities of daily living (ADLs) [5].

Beyond conventional RGB and wearable sensing, radar and LiDAR provide complementary non-wearable capabilities for indoor human monitoring. Millimetre-wave radar can estimate range, radial velocity, and motion-related signatures while being comparatively insensitive to visible-light conditions. Radar–camera fusion has also demonstrated the value of combining complementary geometric, motion, and visual information in human tracking applications [6]. Radar-based sensing may therefore offer advantages in low-light and privacy-sensitive environments, although detailed interpretation of complex human posture can remain challenging. LiDAR, by contrast, provides direct geometric and distance information and can support non-contact indoor activity monitoring without conventional RGB imagery. Its deployment involves different trade-offs relating to line of sight, occlusion, spatial coverage, sensor placement, and environmental integration [7].

These complementary sensing technologies illustrate the broader value of combining modalities that observe different characteristics of human movement.

Despite considerable progress, automatic fall detection systems continue to face an important trade-off between sensitivity and false-alarm suppression. Systems configured to maximise sensitivity may generate false alarms during normal activities such as rapidly sitting down, bending, kneeling, or intentionally lying on the floor [8]. Conversely, conservative decision criteria intended to suppress false alarms may miss genuine falls, particularly slow collapses or falls occurring during transitional movements. Achieving high fall sensitivity while maintaining a low false-positive rate therefore remains a significant challenge.

One reason for this trade-off is that individual sensing modalities observe only part of the physical characteristics of a fall. Vision-based systems can estimate body posture and movement but may become less reliable under poor illumination, occlusion, or challenging viewpoints. Wearable sensors depend on long-term user adherence. Floor-based load-sensing systems provide evidence of physical interaction with the monitored floor, including changes in load distribution and impact-related responses, but floor sensing alone provides limited information about body posture and may not distinguish a person contacting the floor from other load-producing events. Consequently, multimodal approaches that combine complementary sensing information provide a promising means of improving robustness.

The present work investigates such complementarity by combining RGB-derived skeletal motion with distributed floor-load sensing. The vision subsystem provides information about body posture, torso orientation, and temporal movement, whereas the load-sensing platform provides independent evidence of physical interaction with the monitored floor, including impact-related load changes and spatial load distribution. The two modalities therefore observe different physical characteristics of the same event. Rather than relying on either source independently, the proposed framework integrates their decisions within a temporal fusion model to distinguish falls from fall-like ADLs.

Recent advances in deep learning have enabled real-time human pose estimation using lightweight architectures such as YOLOv8 [9]. Unlike conventional object-detection approaches based primarily on bounding boxes, skeletal pose estimation provides information about body configuration, joint locations, and postural dynamics. In the proposed system, YOLOv8n-pose is used as an established pose-estimation component rather than being presented as the principal methodological contribution. The contribution instead lies in combining RGB-derived skeletal evidence with distributed floor-load sensing and temporal decision logic. This combination is intended to exploit complementary visual and physical evidence while retaining a non-wearable architecture, and local processing can reduce the exposure of captured image data.

### 1.2. Related Work and Research Gap

Automatic fall detection has attracted considerable research attention over the past two decades, resulting in a wide range of wearable, vision-based, ambient sensing, and multimodal approaches. Wearable systems based on accelerometers and gyroscopes have demonstrated high sensitivity by detecting characteristic acceleration and orientation changes during falls. However, their performance depends on continuous user compliance, correct device placement, and regular maintenance, making them less suitable for long-term monitoring of older adults and individuals with cognitive impairment [3].

Vision-based methods have evolved from conventional image processing techniques to modern deep learning approaches capable of recognising human activities directly from image sequences. Early systems relied on silhouette extraction, bounding-box aspect ratios, optical flow, or handcrafted geometric features to distinguish falls from normal activities. More recently, convolutional neural networks and human pose estimation models have improved recognition performance by extracting semantic representations of human posture and movement [4]. Among these approaches, YOLOv8-based pose estimation provides an attractive balance between computational efficiency and localisation accuracy by simultaneously detecting human subjects and estimating anatomical keypoints in real time [9]. Compared with conventional bounding-box methods, skeletal pose estimation provides a richer representation of human posture and movement, including body orientation, joint configuration, relative body position, and limb dynamics, which are particularly valuable for recognising fall events.

Ambient sensing approaches offer an alternative privacy-preserving solution by monitoring environmental changes associated with falls. Pressure-sensitive flooring, vibration sensors, acoustic sensors, and radio-frequency sensing have demonstrated promising results without requiring wearable devices or continuous image recording [5]. Nevertheless, these systems generally lack sufficient contextual information regarding human posture and movement, making it difficult to distinguish falls from other high-impact events such as dropped objects or abrupt furniture movement.

Despite these advances, existing fall detection systems continue to face a fundamental challenge in balancing detection sensitivity with false-alarm reduction. Vision-based approaches frequently generate false alarms when normal activities of daily living—such as bending, kneeling, rapidly sitting down, or intentionally lying on the floor—exhibit motion characteristics similar to accidental falls [8]. Conversely, approaches employing conservative detection thresholds to minimise false positives may fail to recognise genuine falls, particularly slow collapses or transitional movements. This trade-off remains one of the principal challenges preventing widespread deployment of automatic fall detection systems in residential and assisted-living environments.

Recent research has increasingly explored multimodal sensor fusion to exploit the complementary strengths of different sensing technologies. Data fusion can be performed at the signal, feature, or decision level, each presenting different computational requirements and robustness characteristics [10,11]. Decision-level fusion is particularly attractive because each sensing modality can operate independently while contributing complementary evidence to the final decision, improving robustness against sensor failure, environmental variability, and measurement uncertainty.

Although multimodal approaches have demonstrated improved performance, relatively few studies have combined modern real-time skeletal pose estimation with distributed floor-based force sensing using a decision-level fusion strategy. Furthermore, many existing systems employ computationally demanding pose estimation frameworks, expensive sensing hardware such as LiDAR, or require wearable devices that reduce user acceptance. Limited attention has also been given to integrating temporal reasoning with complementary physical evidence obtained from floor-based force and load measurements, despite the potential of such information to reduce false alarms associated with normal daily activities.

These limitations reveal an important research gap: There remains a need for a practical, low-cost fall detection framework capable of combining accurate skeletal posture estimation with floor-based force sensing while maintaining real-time performance and high detection reliability. Addressing this gap motivates the development of the multimodal sensor fusion framework presented in this study. To further position the proposed framework within the current state of the art, Table 1 summarises representative fall detection approaches and compares them with the proposed system. The comparison highlights the sensing modality, pose estimation capability, sensor fusion strategy, real-time suitability, and principal limitations of each approach. It demonstrates that while many studies have investigated individual sensing modalities, relatively few have combined real-time skeletal pose estimation with distributed floor-based force sensing using a decision-level fusion framework.

Because the studies employ different participant populations, datasets, activity definitions, train–test partitions, and evaluation metrics, their reported numerical performance should not be interpreted as a direct ranking. The comparison is intended to position the sensing and algorithmic characteristics of the proposed framework, rather than to claim superiority across datasets (see Table 2).

### 1.3. Proposed Approach

To address the limitations of existing single-modality fall detection systems, this study proposes a real-time, non-wearable multimodal fall detection framework that combines vision-based skeletal pose estimation with floor-based force sensing through a decision-level sensor fusion strategy. The proposed framework integrates complementary information from both sensing modalities to improve detection robustness while maintaining low computational complexity and supporting privacy-conscious local processing for deployment in residential care environments.

The vision subsystem employs an Intel RealSense D435 RGB-D camera together with the YOLOv8n-pose network to continuously estimate human skeletal keypoints. Unlike conventional fall detection methods that rely primarily on bounding-box geometry, the proposed approach exploits anatomical keypoint locations to derive skeletal postural and motion descriptors. These include torso inclination, body orientation, normalised trunk-centroid position, trunk-centroid velocity, and postural transitions, providing a more informative representation of fall dynamics than simple silhouette or bounding-box analysis [9,15].

In parallel, a distributed floor-mounted load-sensing array continuously monitors relative floor-loading measurements generated during human movement. The floor-based load-sensing subsystem measures total load, impact intensity, and spatial load distribution, providing complementary physical evidence that cannot be inferred reliably from vision alone. While vision-based systems may misclassify intentional lying or rapid sitting as falls, floor-based force sensing can identify characteristic impact patterns associated with genuine fall events. Conversely, floor-based load sensing alone lacks information regarding body posture and movement, making it difficult to distinguish falls from dropped objects or other environmental disturbances [5].

Rather than merging raw sensor measurements or extracted features, the proposed framework performs decision-level fusion by allowing each sensing modality to independently analyse the observed event before combining their outputs through a temporal finite-state decision model. Decision-level fusion offers several advantages, including modular system design, robustness to temporary sensor failure, reduced computational complexity, and improved scalability for future integration of additional sensing modalities [10,11]. Temporal reasoning is incorporated to model the natural sequence of a fall event, enabling the framework to distinguish accidental falls from normal activities of daily living by considering both movement dynamics and the persistence of the final posture.

The contribution of this study is not the introduction of a new pose-estimation network or a novel general-purpose fusion algorithm. Rather, it lies in the integration and experimental evaluation of complementary vision-derived skeletal features and distributed floor-load measurements within a real-time, decision-level fall detection framework. The evaluation examines the individual and combined contributions of these sensing modalities under a controlled trial protocol, including their respective detection performance, false-positive behaviour, and temporal response. This provides an implementation-level assessment of the potential benefits and limitations of combining visual evidence of body configuration and motion with physical evidence of floor interaction. The findings are preliminary and specific to the evaluated participants and experimental conditions.

The complete system is implemented within a Robot Operating System 2 (ROS2) architecture, allowing asynchronous communication between sensing modules while supporting real-time processing. The modular design enables each sensing component to operate independently, facilitating future system expansion and straightforward deployment in smart-home and assisted-living environments.

The proposed framework was evaluated using representative indoor fall scenarios and activities of daily living performed by healthy volunteers. Experimental evaluation considered detection sensitivity, specificity, precision, accuracy, F1-score, detection latency, and comparative performance against vision-only and load-only configurations. Through this comprehensive evaluation, this study investigates whether integrating complementary skeletal-pose and floor-load information can reduce false alarms while maintaining reliable fall detection performance in real-time indoor monitoring applications.

### 1.4. Contributions and Paper Organisation

This paper presents a practical multimodal fall detection framework that combines skeletal pose estimation with distributed floor-based force sensing for real-time indoor monitoring. Unlike conventional single-modality approaches, the proposed framework exploits complementary visual postural information and floor-interaction evidence through a decision-level sensor fusion strategy to improve detection robustness while maintaining computational efficiency.

The principal scientific contributions of this work are summarised as follows:

1. A decision-level multimodal sensor fusion framework integrating YOLOv8 skeletal pose estimation with a distributed load-sensing platform for real-time indoor fall detection. The proposed framework combines complementary visual postural information and floor-load evidence to improve detection robustness compared with individual sensing modalities.
2. A temporal finite-state decision model that combines human posture, normalised trunk-centroid motion, torso orientation, impact detection, and spatial load distribution to distinguish accidental falls from activities of daily living while reducing false-positive detections.
3. The design, implementation, and calibration of a low-cost floor-based sensing platform capable of continuously monitoring relative floor-loading measurements, impact events, and load distribution, providing complementary information to vision-based posture estimation without requiring wearable devices.
4. A comprehensive experimental evaluation using representative fall scenarios and activities of daily living performed in a controlled indoor environment configured to represent a residential setting. The proposed framework achieved an F1-score of 0.935, a sensitivity of 90.0%, a specificity of 97.5%, and reduced the false-positive count by 82% compared with a vision-only configuration, while maintaining real-time performance.
5. An experimental comparison among vision-only, load-only, and the proposed multimodal temporal fusion configuration using the same evaluation dataset, quantifying the contribution of complementary floor-sensor corroboration relative to the evaluated single-modality baselines.

The remainder of this paper is organised as follows: Section 2 presents the materials and methods, including the sensing platform, skeletal and floor-sensor feature extraction, temporal fusion framework, experimental protocol, ground-truth labelling, and statistical analysis. Section 3 reports the experimental results, including classification performance, modality comparisons, failure analysis, and computational performance. Section 4 discusses the findings, practical implications, and study limitations. Finally, Section 5 concludes the paper and identifies directions for future research.

## 2. Materials and Methods

### 2.1. Overall System Architecture

**Figure 1:**
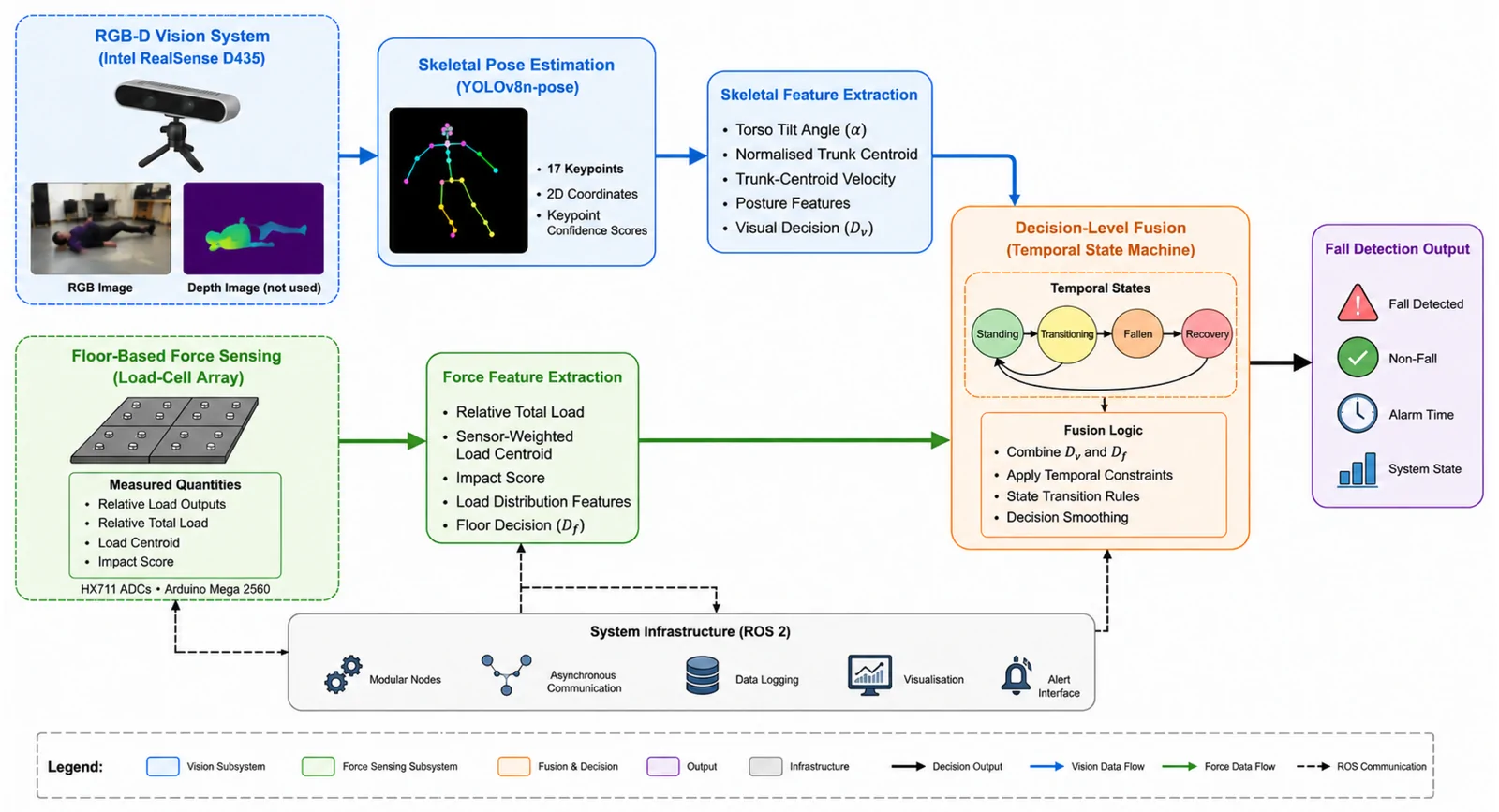
Overall architecture of the proposed multimodal fall detection framework. Images acquired using the Intel RealSense D435 camera are processed using the YOLOv8n-pose network to estimate human skeletal keypoints and derive posture-related skeletal descriptors. Simultaneously, measurements acquired from the distributed load-sensing platform are processed to estimate load-related features, including relative total load, sensor-output-weighted load centroid, and impact intensity. Independent decisions generated by the vision and force-sensing subsystems are subsequently combined using a temporal decision-level fusion module to classify activities as falls or activities of daily living (ADLs).

The proposed multimodal fall detection framework combines vision-based skeletal pose estimation with floor-based force sensing through a decision-level sensor fusion strategy. The system is non-wearable and supports privacy-conscious local processing; however, the use of RGB imagery means that privacy remains an important consideration for real-world deployment. An overview of the proposed architecture is presented in Figure 1.

The framework consists of three principal modules: (i) an RGB-D vision subsystem responsible for estimating human skeletal posture, (ii) a floor-based sensing subsystem that monitors floor-load response and impact events using a distributed load-sensing array, and (iii) a decision-level fusion module that combines the outputs of both sensing modalities to determine whether a fall has occurred.

The vision subsystem acquires image data using an Intel RealSense D435 RGB-D camera. In the implementation evaluated in this study, the RGB stream is processed using the YOLOv8n-pose network to estimate human skeletal keypoints. These two-dimensional keypoint coordinates are subsequently used to derive image-based skeletal descriptors, including torso inclination, normalised trunk-centroid position, and trunk-centroid velocity.

Although synchronised depth measurements are available from the Intel RealSense D435, depth information was not directly incorporated into the fall-classification algorithm evaluated in this study. This was a deliberate design choice to isolate the complementary contribution of two sensing domains: RGB-derived skeletal posture and motion features, and direct floor-interaction information obtained from the distributed load-sensing platform. Accordingly, the D435 was used primarily as the image acquisition platform for the YOLOv8n-pose pipeline rather than as a source of three-dimensional features.

The exclusion of depth should nevertheless be regarded as a limitation of the present implementation rather than an indication that depth information is uninformative. Depth-derived descriptors could provide camera-relative three-dimensional trunk displacement, subject-to-camera distance, and additional geometric information that is not recoverable from two-dimensional image coordinates alone. Such information may improve robustness under viewpoint variation, partial body overlap, self-occlusion, and ambiguous 2D postures. Future extensions will therefore investigate RGB-D skeletal features within the same modular fusion architecture and quantify their incremental contribution relative to the present RGB–load-sensing configuration.

In parallel, the floor-based load-sensing subsystem continuously acquires relative load measurements from the distributed load-sensing array. The acquired signals are processed to estimate the total applied load, spatial load distribution, load centroid, and impact intensity. These load-based features provide complementary information regarding human interaction with the floor surface and enable the identification of high-impact events associated with falls.

Rather than combining heterogeneous sensor measurements directly, the proposed framework adopts a decision-level fusion strategy. Independent decisions generated by the vision and force-sensing subsystems are integrated using a temporal finite-state model that exploits the complementary strengths of both sensing modalities. This approach improves robustness to temporary sensor degradation, simplifies system modularity, and allows each sensing modality to operate independently while contributing evidence towards the final fall detection decision.

The complete processing pipeline begins with concurrent acquisition of RGB-D images and load-sensing measurements. Sensor-specific features are extracted independently before being evaluated by their respective detection modules. The resulting decisions are subsequently fused within the temporal decision model, producing the final classification of each activity as either a fall or an activity of daily living (ADL) (see Table 3).

The overall decision generated by the proposed framework is obtained by combining the independent outputs of the vision and floor-based load sensing according to

(1) $$D\left(t\right)=f\left(D_{v}\left(t\right),D_{f}\left(t\right)\right),$$

where $D_{v}\left(t\right)$ and $D_{f}\left(t\right)$ denote the decisions generated by the vision and force-sensing subsystems at time *t*, respectively, and f(·) represents the temporal decision-level fusion function.

### 2.2. Hardware Platform

**Figure 2:**
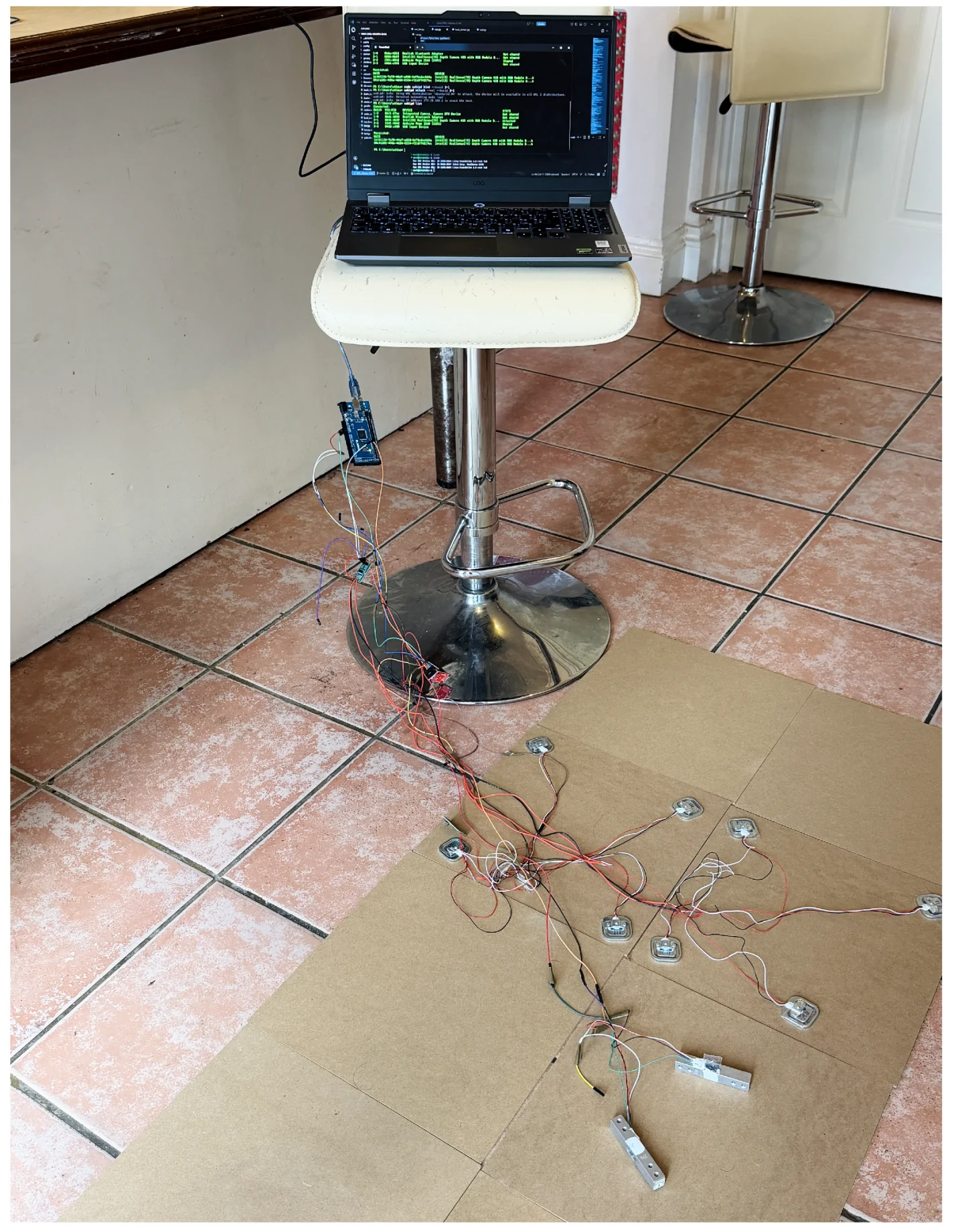
Experimental setup showing the Intel RealSense D435 camera mounted above the monitored area together with the floor-mounted sensing platform used during data collection.

The proposed multimodal fall detection framework integrates complementary vision and floor-based sensing technologies within a unified real-time monitoring platform. The hardware configuration consists of three principal components: (i) an RGB-D vision subsystem for skeletal pose estimation, (ii) a custom-built floor-mounted load-sensing platform for monitoring floor-load response, and (iii) a workstation responsible for real-time sensor processing and decision-level fusion. The complete experimental configuration is illustrated in Figure 2.

The vision subsystem continuously captures synchronised RGB-D images that are processed using the YOLOv8n-pose network to estimate human skeletal posture. Simultaneously, the floor-sensing subsystem measures dynamic loading generated during human movement and extracts complementary load-related features including total load, sensor-output-weighted load centroid, and impact intensity.

Data from both sensing modalities are processed independently before being combined through the proposed decision-level fusion framework. Communication between sensing modules is implemented using the Robot Operating System 2 (ROS2), providing modularity, asynchronous data acquisition, and straightforward integration of additional sensing modalities (see Table 4).

#### 2.2.1. RGB-D Vision System

Human movement was monitored using an Intel RealSense D435 RGB-D camera. The camera simultaneously acquires synchronised RGB and depth images at a spatial resolution of 640 × 480 pixels and a frame rate of 30 frames per second. During all experiments, the camera was mounted approximately 1.9 m above the floor with a downward inclination to maximise coverage of the monitored area while minimising self-occlusion.

RGB images acquired by the Intel RealSense D435 were processed using the YOLOv8n-pose network for real-time skeletal pose estimation. Although the D435 provides synchronised RGB and depth streams, the fall detection algorithm evaluated in the present study uses the RGB stream for pose estimation and derives the proposed skeletal descriptors from two-dimensional image-plane keypoint coordinates. The depth stream was available through the acquisition platform but was not used directly in the decision-level fusion algorithm evaluated in this study. Accordingly, the vision-derived features reported in this work should be interpreted as image-based postural descriptors rather than metric three-dimensional biomechanical measurements. The camera was selected because of its real-time image acquisition capability, compact form factor, and ROS2 compatibility [16] (see Table 5).

#### 2.2.2. Floor-Based Force-Sensing Platform

**Figure 3:**
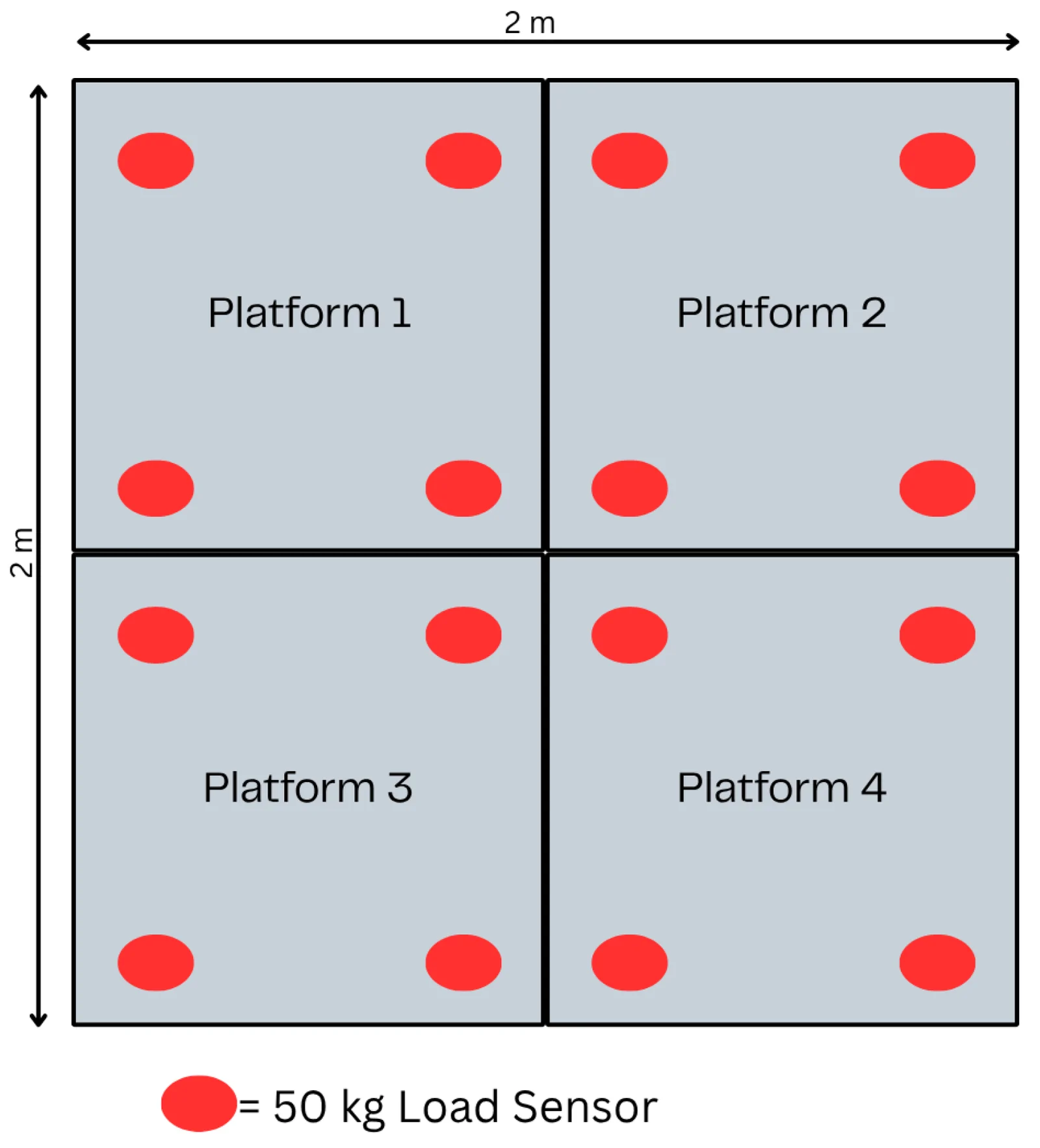
Full arrangement of sensing platform.

Floor-interaction information was obtained using a custom-built distributed load-sensing platform consisting of four adjacent instrumented platforms. Each platform measured approximately 1.0m×1.0m, providing a total instrumented floor area of approximately $4.0\;m^{2}$. The four platforms were arranged as a 2×2 floor array, giving an overall instrumented region of approximately 2.0m×2.0m. The platforms were constructed from solid MDF sheets with a thickness of approximately 8mm. Each 1.0 m × 1.0 m platform incorporated four load cells, each with a rated capacity of 50 kg, positioned near its four corners. The load cells were inset from the physical platform corners by a small padding distance to ensure that loads applied near the platform boundaries were transferred reliably to the sensing elements. Thus, the complete floor-sensing system comprised 16 load cells distributed across four independent sensing channels, with four load cells associated with each platform (see Figure 3).

**Figure 4:**
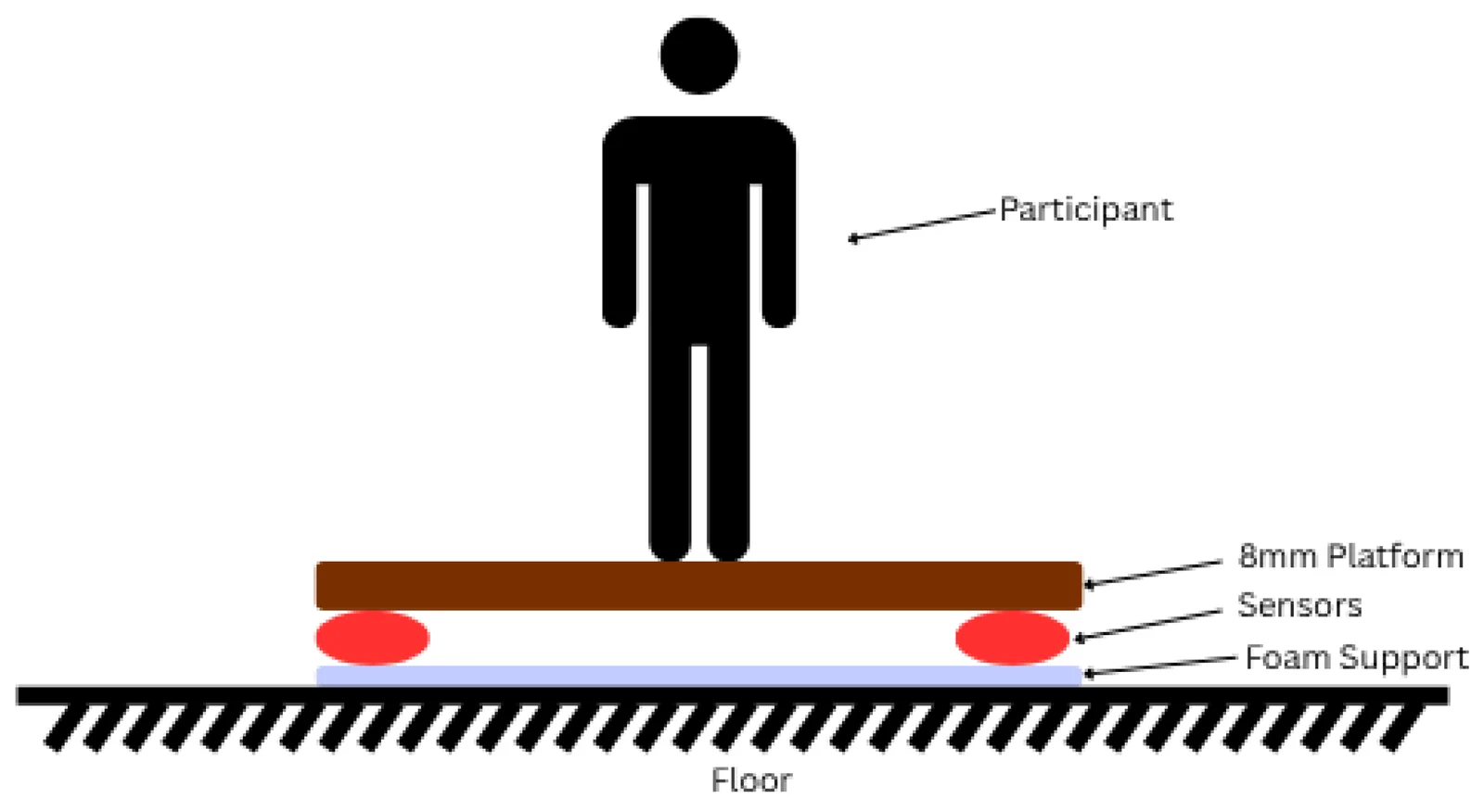
Structure arrangement diagram.

The load-transfer structure was supported using packing foam beneath the sensing elements, maintaining the load cells above the underlying floor and allowing the applied load on the MDF platform to be transferred through the sensing elements. The resulting arrangement allowed vertical loading caused by a participant standing, walking, or falling on the instrumented platforms to be detected independently across the four floor-sensing regions (see Figure 4).

The load-cell bridge outputs were interfaced with HX711 instrumentation-amplifier and analogue-to-digital converter modules. The HX711 modules provided signal amplification and high-resolution digitisation before the measurements were acquired by the Arduino Mega 2560.

Because the instrumented floor region covered a finite $4.0\;m^{2}$ area while the camera monitored a larger field of view, falls occurring partially or completely outside the instrumented region could provide insufficient floor-loading evidence for multimodal confirmation.

This geometric limitation is reflected in the experimental failure analysis, where four false-negative fall trials occurred when the participant landed partially or wholly outside the 2.0m×2.0m instrumented region.

#### 2.2.3. Processing Hardware

Real-time processing was performed on a workstation running Ubuntu 24.04 LTS through Windows Subsystem for Linux 2 (WSL2), equipped with an AMD Ryzen 7 7435HS processor (3.10 GHz), an NVIDIA GeForce RTX 4060 Laptop GPU with 8 GB of dedicated graphics memory, and 16 GB of installed system memory. Vision processing, skeletal feature extraction, force sensing, and decision-level fusion were implemented within the Robot Operating System 2 (ROS 2) environment. The reported real-time performance therefore corresponds specifically to this workstation configuration. The present study did not evaluate deployment on a resource-constrained edge computing platform; consequently, performance on embedded or edge hardware is not claimed. Evaluation on such platforms remains an important direction for future work.

The modular processing architecture enables each sensing modality to operate asynchronously, facilitating future integration of additional sensing technologies without modification of the existing process pipeline.

### 2.3. Vision-Based Skeletal Pose Estimation

The vision subsystem continuously estimates human posture from RGB images acquired using the Intel RealSense D435 camera. Human skeletal pose estimation is performed using the YOLOv8n-pose network, which simultaneously detects human subjects and predicts anatomical keypoints in real time. Compared with conventional fall detection approaches based solely on bounding-box geometry, skeletal pose estimation provides a richer representation of body posture by explicitly estimating anatomical keypoint locations. These descriptors include torso inclination, body orientation, normalised trunk-centroid position, and trunk-centroid velocity, which together provide a robust representation of postural transitions associated with both falls and activities of daily living.

#### 2.3.1. YOLOv8 Pose Estimation

Human skeletal pose estimation was performed using the YOLOv8n-pose architecture [9]. The network simultaneously performs human detection and anatomical keypoint localisation using a single-stage deep learning framework, supporting real-time skeletal pose estimation.

For each detected subject, the network predicts a bounding box together with seventeen anatomical keypoints corresponding to the major joints of the human body. Each detected keypoint is represented by its image coordinates and an associated confidence score, which quantifies localisation reliability. Only keypoints exceeding a predefined confidence threshold are retained for subsequent feature extraction. Each YOLOv8n-pose inference produces detected persons $P=\{p_{1},p_{2},\ldots ,p_{N}\}$. For each detected person, the model estimates a bounding box (x,y,w,h), a detection confidence $c_{i}\in \left[0,1\right]$, and K=17 skeletal keypoints $k_{j}=\left(u_{j},v_{j},s_{j}\right)$, where $\left(u_{j},v_{j}\right)$ denote image coordinates and $s_{j}$ represents the corresponding keypoint confidence.

The observed skeletal keypoints are modelled as

(2) $$k_{j}^{obs}=k_{j}^{true}+\epsilon_{j},\;\epsilon_{j}∼N\left(0,\sigma_{j}^{2}I\right),$$

where $\epsilon_{j}$ represents localisation noise arising from image uncertainty, partial occlusion, and detector variability. Keypoints with confidence values below the threshold given in Table 6 were discarded prior to feature extraction.

#### 2.3.2. Skeletal Feature Extraction

The estimated skeletal keypoints were used to derive a set of image-based postural and motion descriptors representing body configuration and movement throughout each activity. Unlike conventional approaches that rely solely on body orientation or bounding-box geometry, the proposed framework combines multiple complementary posture descriptors to improve robustness under varying movement conditions.

The torso inclination angle provides a direct measure of upper-body orientation relative to the vertical axis. Increasing torso inclination is typically associated with the transition from an upright posture towards a fall event.

(3) $$\alpha =\arctan\left(\frac{u_{neck}-u_{hip}}{v_{hip}-v_{neck}}\right)$$

The torso inclination angle was used as one of the posture descriptors within the decision-level fusion framework. Based on empirical evaluation during the pilot experiments, three posture regions were defined: torso inclination angles below $30^{\circ }$ were classified as upright, angles between $30^{\circ }$ and $60^{\circ }$ were considered transitional, and angles greater than $60^{\circ }$ were classified as lying or near-horizontal. These thresholds were selected to provide reliable discrimination among normal standing activities, transitional movements, and fall-related postures while maintaining robustness across participants.

(4) $$P_{\alpha }=\left\{\begin{array}{cc} \mathrm{Standing}, & |\alpha |<30^{\circ },\\ \mathrm{Transition}, & 30^{\circ }\leq \left|\alpha \right|<60^{\circ },\\ \mathrm{Lying}, & \left|\alpha \right|\geq 60^{\circ }, \end{array}\right.$$

where $\left(u_{neck},v_{neck}\right)$ and $\left(u_{hip},v_{hip}\right)$ represent the image coordinates of the neck and hip midpoints, respectively. Larger absolute values of α indicate increasingly horizontal body posture.

A normalised image-plane trunk-centroid position was estimated using the four principal trunk keypoints corresponding to the left and right shoulders and hips. This descriptor provides a two-dimensional representation of vertical trunk position and is used to characterize body movement during standing, walking, sitting, and falling activities.

(5) $$y_{\mathrm{trunk}}\left(t\right)=\frac{1}{H}\frac{\sum_{j\in J_{\mathrm{core}}}s_{j}v_{j}}{\sum_{j\in J_{\mathrm{core}}}s_{j}},$$

where $J_{\mathrm{core}}$ denotes the set of left and right shoulder and hip keypoints, $s_{j}$ is the corresponding keypoint confidence, $v_{j}$ is the vertical image coordinate of keypoint *j*, and *H* is the image height. Normalisation by *H* produces a dimensionless image-plane vertical-position descriptor.

Rapid downward movement is quantified by differentiating consecutive trunk-centroid estimates:

(6) $${\dot{y}}_{\mathrm{trunk}}\left(t\right)=\frac{y_{\mathrm{trunk}}\left(t\right)-y_{\mathrm{trunk}}\left(t-\Delta t\right)}{\Delta t},$$

where Δt corresponds to the image acquisition interval. A value of ${\dot{y}}_{\mathrm{trunk}}\left(t\right)$ exceeding the threshold listed in Table 7 indicates rapid downward image-plane motion consistent with a potential fall. Since the position descriptor is normalised by image height, ${\dot{y}}_{\mathrm{trunk}}$ represents normalised image-plane velocity rather than metric physical velocity in $m\;s^{-1}$.

The extracted skeletal descriptors constitute the visual evidence used by the proposed decision-level fusion framework. These posture-related features are subsequently combined with complementary load and floor-interaction information obtained from the load-sensing platform to improve fall detection robustness while reducing false-positive detections during activities of daily living.

### 2.4. Floor-Based Force Sensing

The floor-sensing subsystem provides complementary physical information by continuously monitoring load changes generated during human interaction with the instrumented floor surface. Unlike vision-based sensing, which primarily characterises body posture, the distributed load-sensing platform directly provides relative measurements of physical interaction between the participant and the instrumented floor surface. The sensing platform consists of a distributed array of load cells mounted beneath a rigid monitoring surface. Each load cell produces an electrical signal related to the local load transferred through the instrumented surface. The load-cell outputs are conditioned and digitised using HX711 amplifier/analogue-to-digital converter modules before transmission to the Arduino Mega 2560.

- Total load;
- load centroid $C_{L}$;
- Impact score.

These descriptors provide complementary evidence regarding floor interaction during both falls and activities of daily living, improving robustness when combined with visual posture information. The total load is computed by summing the outputs of all active sensors,

(7) $$L_{\mathrm{total}}\left(t\right)=\sum_{i=1}^{N_{s}}V_{i}^{\mathrm{cal}}\left(t\right),$$

where $V_{i}^{\mathrm{cal}}\left(t\right)$ denotes the calibrated relative output of the *i*-the load sensor, and $N_{s}$ is the total number of sensors. The spatial distribution of the applied load is represented by the sensor-output-weighted load centroid,

(8) $$C_{L}\left(t\right)=\frac{\sum_{i=1}^{N_{s}}V_{i}^{\mathrm{cal}}\left(t\right)\left(x_{i},y_{i}\right)}{\sum_{i=1}^{N_{s}}V_{i}^{\mathrm{cal}}\left(t\right)},$$

where $\left(x_{i},y_{i}\right)$ denotes the spatial location of the *i*-th sensor within the instrumented floor area. Because the weighting is based on calibrated relative load-sensor outputs rather than absolute force measurements, $C_{L}\left(t\right)$ is interpreted as a voltage-weighted load-centroid descriptor rather than a biomechanical centre-of-pressure measurement.

(9) $$I\left(t\right)=\frac{L_{\mathrm{total}}\left(t\right)-L_{\mathrm{total}}\left(t-\Delta t\right)}{\Delta t}$$

where Δt is the load-sensor sampling interval. An impact score exceeding the threshold listed in Table 6 is interpreted as evidence of floor impact (see Table 8).

### 2.5. Load Sensor Calibration

Accurate and repeatable load measurements require calibration of the load-sensing platform to compensate for manufacturing variability and nonlinear sensor response. Prior to experimental evaluation, each load sensor was calibrated individually using a series of known static reference loads.

Known calibration masses were sequentially applied directly above each sensing element while the corresponding analogue voltage output was recorded. Measurements were repeated three times for each load level, and the mean response was used to characterise the sensor behaviour.

Because the proposed framework relies primarily on relative load-based descriptors rather than absolute force estimation, calibration was performed to improve consistency between sensors rather than to obtain high-precision force reconstruction. The resulting calibration coefficients were subsequently applied during online processing to compensate for sensor-to-sensor variability. The calibrated sensor output is expressed as

(10) $$V_{i}^{\mathrm{cal}}=a_{i}V_{i}+b_{i}$$

where $a_{i}$ and $b_{i}$ represent the calibration coefficients determined for the *i*-th sensor.

### 2.6. Decision-Level Sensor Fusion

The proposed framework adopts a decision-level sensor fusion strategy to combine complementary information obtained independently from the vision and floor-sensing subsystems. Unlike signal-level or feature-level fusion, decision-level fusion allows each sensing modality to operate independently while contributing evidence towards the final classification decision. The fall-classification stage itself is a deterministic, rule-based temporal decision framework rather than a learned classifier. YOLOv8n-pose is used only to provide the skeletal observations required by the vision branch. The vision branch converts the extracted skeletal descriptors into a binary visual transition decision $D_{v}\left(t\right)$, while the floor-sensing branch converts the load-derived descriptors into a binary floor-event decision $D_{f}\left(t\right)$. These modality-specific decisions, together with their temporal histories, are evaluated by the finite-state model.

The overall fusion process is expressed as

(11) $$D\left(t\right)=f\left(D_{v}\left(t\right),D_{f}\left(t\right)\right),$$

where $D_{v}\left(t\right)$ and $D_{f}\left(t\right)$ represent the vision-based and force-based decisions at time *t*, respectively.

A fall decision is not generated from a single-frame conjunction of the two sensing modalities. Instead, the proposed framework integrates temporally accumulated visual and force-based evidence within the finite-state decision model. The vision subsystem identifies a candidate fall transition when rapid downward trunk-centroid motion, increasing torso inclination, or another substantial postural change is detected. A fall is subsequently confirmed only when the temporal sequence of observations satisfies the transition and persistence criteria defined by the state machine.

Accordingly, the fused fall decision can be expressed as

(12) $$D\left(t\right)=\left\{\begin{array}{cc} \mathrm{Fall}, & T_{v}\left(t\right)\wedge E_{f}\left(t\right)\wedge P_{l}\left(t\right),\\ \mathrm{Non-Fall}, & \mathrm{otherwise}, \end{array}\right.$$

where $T_{v}\left(t\right)\in \left\{0,1\right\}$ denotes whether visual evidence of an upright-to-floor transition has been detected within the fall window $T_{f}$, $E_{f}\left(t\right)\in \left\{0,1\right\}$ denotes whether corroborating floor-sensor evidence has been detected, and $P_{l}\left(t\right)\in \left\{0,1\right\}$ denotes whether the required floor-level or lying-posture persistence criterion has been satisfied. These criteria are evaluated over a temporal interval and are therefore not required to occur simultaneously within a single image frame. Consequently, the fusion rule represents the logical evidence required for fall confirmation, whereas the temporal ordering of these observations is governed by the finite-state model described in the following subsection.

### 2.7. Temporal State Machine

To improve robustness against transient posture changes and isolated sensor events, the fused observations are evaluated using a temporal finite-state machine. Rather than relying on instantaneous measurements, the state machine models the sequential evolution of posture, motion, and floor-interaction evidence throughout a potential fall event.

Four states are defined:

- Standing;
- Transitioning;
- Fallen;
- Recovery.

The system normally remains in the Standing state while the subject exhibits an upright posture without substantial downward trunk-centroid motion. A transition to the Transitioning state occurs when rapid downward trunk-centroid motion, increasing torso inclination, or another substantial postural change provides visual evidence of a potential fall.

During the Transitioning state, the system searches for corroborating evidence from the load-sensing platform, including an impact event or a load-distribution pattern consistent with body–floor contact. The candidate fall transition must occur within the maximum fall interval $T_{f}$. If the required evidence is not observed within this interval, the event is rejected and the system returns to the Standing state.

Following floor contact, the system evaluates whether the subject remains in a floor-level or lying configuration for at least the minimum persistence duration $T_{l}$. Once the required visual transition evidence, floor-sensor corroboration, and postural persistence have been observed, the system enters the Fallen state and generates a fall alarm. Importantly, the individual criteria are not required to occur simultaneously within a single frame; rather, evidence is accumulated over the fall sequence.

The Fallen state is maintained while the subject remains in the detected floor-level posture. When the visual subsystem indicates a sustained transition away from the floor-level configuration towards an upright posture, the system enters the Recovery state. Once an upright posture is re-established, the state machine returns to Standing and resumes monitoring for subsequent events.

The temporal state evolution can be represented as

(13) $$S\left(t+\Delta t\right)=g\left(S\left(t\right),V_{0:t},F_{0:t},\Delta T\right),$$

where S(t) denotes the current system state, $V_{0:t}$ and $F_{0:t}$ represent the recent histories of vision-derived and floor-sensor observations, respectively, and ΔT denotes the elapsed time since entry into the current state. The transition function g(·) evaluates the accumulated observations together with the temporal constraints $T_{f}$ and $T_{l}$ to determine the subsequent state.

This temporal reasoning reduces false alarms caused by transient activities such as rapid sitting, kneeling, bending, or intentional lying by requiring an appropriate sequence of postural transition, floor-interaction evidence, and sustained floor-level posture before a fall is confirmed.

### 2.8. Decision Thresholds

The proposed fall detection framework employs a small number of empirically selected thresholds to distinguish fall events from activities of daily living. These parameters were determined during preliminary experiments using a separate pilot dataset collected prior to the main evaluation and were subsequently fixed for all experimental trials. Table 6 summarises the thresholds used by the proposed framework.

The decision thresholds were determined empirically during preliminary experiments using pilot trials collected before the main evaluation. The pilot trials were excluded from the 160-trial evaluation dataset; however, the pilot and evaluation trials involved the same pool of eight participants. Accordingly, the separation was at the trial level rather than the participant level. Candidate threshold values were examined during preliminary testing to establish an operating point that balanced fall sensitivity against false-positive detections. The selected thresholds were subsequently fixed and applied unchanged throughout the main evaluation. This procedure avoids adjusting the thresholds using the main evaluation trials, but it does not establish generalisation to previously unseen participants. The selected values should therefore be interpreted as empirically determined operating parameters for the evaluated experimental conditions.

To further clarify the expected sensitivity of the decision logic to threshold selection, the principal thresholds were considered qualitatively in terms of their effects on fall detection and false-alarm behaviour. The torso-angle thresholds define the transition between upright, uncertain, and near-horizontal postures; reducing the lying-posture threshold would make the classifier more responsive to less-horizontal postures, but it could increase the likelihood of incorrectly classifying transitional or non-fall activities as lying. A more conservative threshold would reduce this susceptibility but could delay or prevent classification of falls that do not result in a strongly horizontal posture.

Similarly, the trunk-centroid velocity threshold controls the sensitivity to rapid downward motion. A lower threshold would allow slower downward movements to contribute to candidate-fall detection, while potentially increasing sensitivity to non-fall movements. Conversely, a higher threshold would require more pronounced downward motion and could reduce false detections at the expense of potentially missing slower fall events. The impact threshold provides an analogous trade-off for the floor-sensing modality: lowering it would increase responsiveness to lower-magnitude impacts but could admit more non-fall load changes, whereas increasing it would provide more conservative impact detection and could reduce sensitivity to gentle or slow-collapse events.

The temporal persistence threshold $T_{l}$ introduces a further sensitivity–delay trade-off. A shorter persistence requirement would permit earlier confirmation of a candidate fall but would provide less temporal separation between genuine falls and brief floor-level activities. Increasing $T_{l}$ would provide stronger temporal confirmation but would necessarily increase the delay before the final alarm. These effects represent the expected behaviour of the decision logic rather than additional experimental measurements.

Accordingly, the reported performance should be interpreted as the performance of the fixed operating point selected from the preliminary pilot evaluation, rather than as evidence that the classifier is insensitive to threshold variation. Formal threshold sweeps and validation across different participants, camera viewpoints, room configurations, and fall dynamics remain important directions for future work.

### 2.9. Detection Latency

The temporal architecture of the proposed framework involves two distinct time points following fall onset that must be carefully distinguished:

The **candidate-fall detection time** $T_{\mathrm{candidate}}$ denotes the moment the fusion node first accumulates sufficient evidence to identify a potential fall transition—specifically, when rapid downward trunk-centroid motion and increasing torso inclination satisfy the visual transition criterion and are corroborated by a positive load-sensor impact event. At this point, the system enters the Transitioning state. No alarm is issued yet.

The **final alarm time** $T_{\mathrm{alarm}}$ denotes the moment the system enters the Fallen state and publishes the fall alarm. This occurs only after the floor-level posture has been sustained for the minimum persistence duration $T_{l}=2.0$ s following $T_{\mathrm{candidate}}$. Therefore, by construction,

(14) $$T_{\mathrm{alarm}}=T_{\mathrm{candidate}}+T_{l}+\delta ,$$

where δ≥0 accounts for any additional frame-level delay in posture confirmation. The minimum possible alarm latency relative to fall onset is thus $T_{l}=2.0$ s.

The **detection latency** reported in this study is defined as the candidate-fall detection latency:

(15) $$T_{\mathrm{latency}}=T_{\mathrm{candidate}}-T_{\mathrm{onset}},$$

where $T_{\mathrm{onset}}$ is the manually annotated timestamp at which the participant initiated the fall transition from the preceding upright or stable posture. This metric captures how quickly the system identifies the onset of a fall event from sensor evidence, independently of the mandatory persistence confirmation window.

The mean candidate-fall detection latency is calculated as

(16) $${\bar{T}}_{\mathrm{latency}}=\frac{1}{N_{\mathrm{TP}}}\sum_{i=1}^{N_{\mathrm{TP}}}T_{\mathrm{latency},i},$$

where $N_{\mathrm{TP}}$ is the number of true-positive fall trials. Detection latency was calculated only for true-positive fall trials.

This two-stage timing model means that the reported mean candidate-fall detection latency of 1.80±0.41 s is fully consistent with the $T_{l}=2.0$ s persistence requirement: the candidate detection occurs on average 1.80 s after fall onset, after which the system waits a further 2.0 s before issuing the alarm.

The total elapsed time from fall onset to final alarm generation is therefore approximately 3.8 s on average. This value reflects the explicit trade-off introduced by the $T_{l}=2.0$ s persistence criterion and should be interpreted in relation to the false-alarm suppression achieved by the temporal confirmation stage [17].

### 2.10. Experimental Protocol

The proposed multimodal fall detection framework was evaluated under controlled indoor conditions. Eight healthy participants (four male and four female), aged 19–34 years, were recruited from the Anglia Ruskin University staff and student population. Participants reported no musculoskeletal, neurological, or mobility impairments that would affect their ability to perform the experimental activities. Ethical approval was obtained from the Anglia Ruskin University Research Ethics Committee (ETH2526-3122), and written informed consent was obtained from all participants prior to data collection. The activity order was fixed rather than randomised; therefore, potential order or familiarisation effects cannot be excluded. Experimental trials were conducted in an indoor environment designed to represent a residential living space. The monitored area included a tiled floor, domestic furniture, and natural illumination. An Intel RealSense D435 camera was mounted at a height of approximately 1.9 m and inclined downward by approximately $12^{\circ }$. The distributed load-sensing platform was positioned beneath a thin rubber mat within the monitored floor area.

Each participant completed five simulated fall scenarios:

1. Forward fall;
2. Lateral fall;
3. Backward fall;
4. Slow collapse;
5. Stumble fall.

To evaluate false-positive performance, five activities of daily living (ADLs) were also performed:

1. Intentional lying down;
2. Rapid sitting to the floor;
3. Bending to pick up an object;
4. Kneeling followed by returning to standing;
5. Walking through or exiting the monitored area.

Each activity was repeated twice by each participant. Consequently, each participant completed 20 trials, comprising 10 simulated-fall trials and 10 ADL trials. Across the eight participants, the complete evaluation dataset therefore consisted of 160 trials: 80 simulated falls and 80 ADL trials.

Before data collection, participants completed a structured warm-up and received instructions regarding the safe execution of the simulated fall activities. During each trial, the vision and floor-sensing streams were acquired concurrently and timestamped within the ROS2 framework. The participant characteristics are summarised in Table 9.

**Figure 5:**
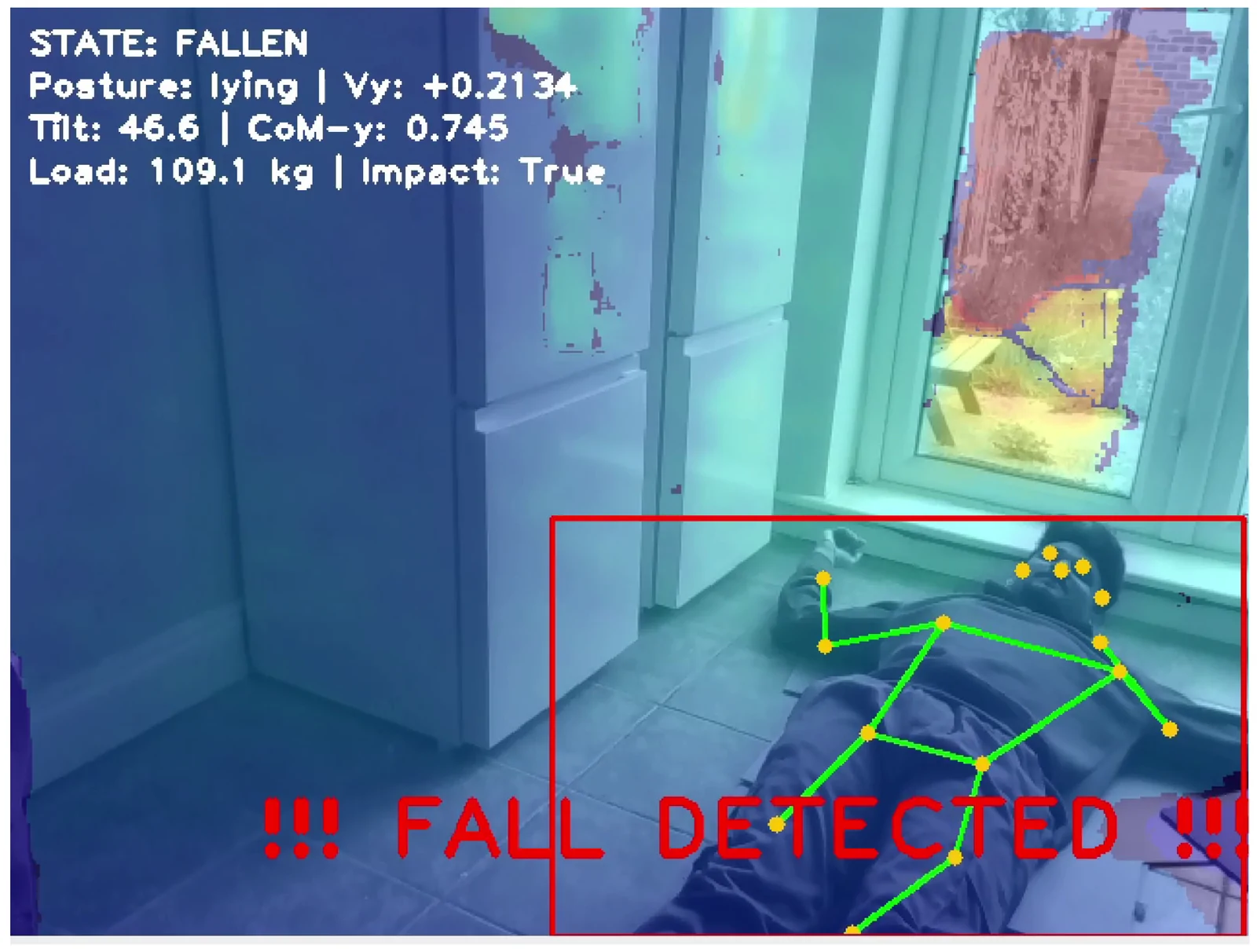
System output during a confirmed fall detection event (Participant P3, forward fall trial). At the displayed frame, the fusion node reports STATE: FALLEN, with normalised trunk-centroid velocity ${\dot{y}}_{\mathrm{trunk}}=+0.2134$, instantaneous torso inclination $46.6^{\circ }$, normalised trunk-centroid position $y_{\mathrm{trunk}}=0.745$, and a positive load-impact flag. The Fallen state reflects temporally accumulated evidence from the preceding fall sequence rather than the instantaneous torso-angle value alone. The labels “CoM-y” and “$V_{y}$” shown in the software overlay correspond to the normalised trunk-centroid position and velocity defined in Equations (5) and (6), respectively.

For real-time operator visualisation, system telemetry was overlaid on the display feed, as illustrated in Figure 5. This visualisation was used for monitoring only and did not alter the RGB-based pose-estimation and decision-fusion pipeline.

#### Ground-Truth Labelling

Each experimental trial was assigned a ground-truth activity label according to the predefined experimental protocol. Trials corresponding to the five simulated fall scenarios were labelled as falls, whereas trials corresponding to the five activities of daily living were labelled as non-fall events.

For temporal evaluation, recorded RGB sequences were reviewed manually to identify the onset of each simulated fall. The ground-truth fall-onset time, $T_{\mathrm{onset}}$, was defined as the timestamp at which the participant initiated the fall transition from the preceding stable or upright posture. This timestamp was used as the temporal reference for calculating detection latency. A fall alarm generated within 5 s of the manually annotated fall onset was associated with that ground-truth event and classified as a true positive. Fall trials without an alarm within this interval were classified as false negatives.

A system output was classified as a true positive (TP) when a fall alarm was generated for a ground-truth fall trial, and as a false negative (FN) when no fall alarm was generated for that trial. For ADL trials, the absence of a fall alarm was classified as a true negative (TN), whereas an incorrectly generated fall alarm was classified as a false positive (FP).

### 2.11. Statistical Analysis

Fall detection performance was evaluated using sensitivity, specificity, precision, accuracy, F1-score, false-positive rate, and detection latency. The classification metrics were calculated from the numbers of true positives (TP), false positives (FP), true negatives (TN), and false negatives (FN).

Sensitivity was calculated as

(17) $$\mathrm{Sensitivity}=\frac{TP}{TP+FN}.$$

Specificity was calculated as

(18) $$\mathrm{Specificity}=\frac{TN}{TN+FP}.$$

Precision was calculated as

(19) $$\mathrm{Precision}=\frac{TP}{TP+FP}.$$

Accuracy was calculated as

(20) $$\mathrm{Accuracy}=\frac{TP+TN}{TP+TN+FP+FN}.$$

The F1-score was calculated as the harmonic mean of precision and sensitivity:

(21) $$F_{1}=2\frac{\mathrm{Precision}\times \mathrm{Sensitivity}}{\mathrm{Precision}+\mathrm{Sensitivity}}.$$

The false-positive rate was calculated as

(22) $$\mathrm{FPR}=\frac{FP}{FP+TN}.$$

For sensitivity, specificity, precision, and accuracy, 95% confidence intervals were estimated using the Wilson score method. This method was selected because it provides more reliable interval estimates for binomial proportions, particularly when the observed proportion is close to zero or one.

Detection latency was calculated for true-positive fall trials according to Equation (15) and is reported as the mean ± standard deviation. Differences in detection latency between fall types were analysed descriptively due to the limited sample size.

### 2.12. Software Implementation

The proposed multimodal fall detection framework was implemented in Ubuntu 24.04 LTS using the Robot Operating System 2 (ROS2) Humble as the middleware for communication and process-management. All processing nodes were written in Python 3.10, with vision processing performed using OpenCV 4 and the Ultralytics YOLOv8 framework. GPU-accelerated inference was enabled through the NVIDIA CUDA Toolkit 12 on the NVIDIA RTX-series GPU of the host workstation.

The pretrained YOLOv8n-pose model was used for inference without task-specific model training. No explicit random seed was configured for the experimental pipeline. The reported results were obtained using the recorded experimental trials and the fixed decision parameters described in Section 2.8.

#### 2.12.1. Node Architecture

The complete processing pipeline is realised as four independent ROS2 nodes communicating over named topics, as summarised in Table 10. This architecture maintains a clear separation of concerns: each node encapsulates a single sensing modality or processing stage, enabling independent development, testing, and future replacement without modifying the remainder of the pipeline.

#### 2.12.2. Vision Processing Node

The pose_estimator_node subscribes to the RGB image topic and performs YOLOv8n-pose inference on each received frame. The YOLOv8n-pose (nano) model variant was selected to balance keypoint localisation accuracy against inference speed, supporting real-time operation at the 30 Hz camera acquisition rate. Inference was configured with a detection confidence threshold of $\tau_{c}=0.50$ and an intersection-over-union threshold of 0.45 for non-maximum suppression. When multiple persons were detected within a single frame, the subject occupying the largest bounding-box area was selected as the primary monitoring target.

For each detected subject, the node extracted the skeletal descriptors defined in Section 2.3.2—torso inclination α, normalised trunk-centroid position $y_{\mathrm{trunk}}$, and trunk-centroid velocity ${\dot{y}}_{\mathrm{trunk}}$—and published them as a custom PoseFeatures message on /pose_features, stamped with the ROS2 system clock for downstream temporal association. Keypoints with confidence below $\tau_{s}=0.50$ were excluded from all feature computations. When fewer than four core trunk keypoints (bilateral shoulders and hips) met this threshold, the posture classification was set to uncertain and velocity features were zeroed to prevent spurious state-machine transitions under heavy occlusion.

A majority-vote filter over a sliding window of W=5 consecutive frames was applied to raw per-frame posture classifications before the state machine received posture updates, suppressing single-frame misclassifications arising from transient detection failures.

#### 2.12.3. Load Sensor Node

The load_sensor_node established a serial connection to the Arduino Mega 2560 at 115,200 baud and received comma-separated digitised load-sensor measurements at 50 Hz. Each received string was parsed, offset-corrected, and sensitivity-normalised using the per-sensor calibration coefficients (Section 2.5). The node then computed the load features defined in Section 2.4—total load $L_{\mathrm{total}}$, sensor-output-weighted load centroid $C_{L}$, and impact score I(t)—and published them as a custom LoadFeatures message on /load_features, stamped with the ROS2 system clock.

To mitigate electrical noise on the Arduino’s analogue input channels, a 10-sample moving-average filter was applied in firmware before serial transmission. At a sampling frequency of 50 Hz, this corresponds to a 200 ms observation window. The filter introduces temporal smoothing and may delay the response to abrupt changes in sensor output. Its contribution to event detection latency was not independently quantified.

#### 2.12.4. Temporal Association in the Fusion Node

The fusion_node subscribed to both /pose_features (30 Hz) and /load_features (50 Hz) simultaneously. Because the two sensing modalities operate at different update rates and communicate asynchronously, strict frame-level synchronisation was neither required nor imposed. Instead, the fusion node maintained an internal cache of the most recently received message from each topic. Upon receipt of any new PoseFeatures message, the node evaluated the current state-machine transition conditions using that message together with the cached LoadFeatures values.

This latest-value association strategy accommodates the asynchronous operation of the two sensing modalities without requiring strict frame-level synchronisation. However, differences in acquisition timing, processing latency, and communication delays may introduce temporal misalignment between the associated measurements. Consequently, the higher sampling frequency of the load-sensing subsystem does not independently guarantee that its measurements are temporally more recent than the corresponding visual observations.

State transitions, feature values, and timestamps were logged continuously to an ROS2 bag file during all experimental sessions, enabling complete post hoc replay and trial-level analysis.

#### 2.12.5. Fall Alarm and Suppression

Upon entering the Fallen state, the fusion node immediately published a stamped Boolean alarm message on /fall_alarm, minimising output latency. A 30 s suppression timer prevented repeated alarms for the same fall event during the post-fall lying period. The current state-machine state was simultaneously published on /system_state as a string message to support real-time operator monitoring and post hoc analysis.

#### 2.12.6. Visualisation

A lightweight visualisation node subscribed to /camera/color/image_raw and /pose_features and overlaid the detected skeleton, posture classification label, current state-machine state, and key feature values on the live RGB feed using OpenCV. This overlay, illustrated in Figure 5, was used for real-time operator monitoring during experimental trials and for diagnostic inspection of detection errors; it did not influence the RGB-based pose estimation or decision-fusion pipeline.

## 3. Results and Analysis

### 3.1. Feature Progression Across Fall Phases

The system’s response during a fall event is illustrated by the representative telemetry shown in Figure 5, captured during a forward fall trial performed by Participant P3. During the standing phase immediately preceding the fall, the torso tilt angle α remained below $30^{\circ }$, the normalised trunk-centroid position $y_{\mathrm{trunk}}$ was approximately 0.35–0.40 (upper portion of the frame), and the load-sensor array registered a relative total-load value of approximately 85 a.u., distributed across a narrow contact footprint consistent with single-stance loading.

During the falling phase, three features responded in rapid succession. The normalised trunk-centroid velocity ${\dot{y}}_{\mathrm{trunk}}$ increased sharply, reaching ${\dot{y}}_{\mathrm{trunk}}=+0.2134\;s^{-1}$ at the frame shown, indicating rapid downward descent. During the transition phase, the torso inclination increased from the upright region (<$30^{\circ }$) into the transitional region ($30^{\circ }$–$60^{\circ }$).

The summed load-sensor response is reported in arbitrary units (a.u.) because the sensing platform was calibrated for relative sensor consistency rather than absolute force reconstruction. Following floor contact, the accumulated visual evidence became consistent with a sustained floor-level posture, with $y_{\mathrm{trunk}}$ reaching 0.745. Concurrently, the relative total-load descriptor stabilised at approximately 109.1 a.u., while the spatial distribution of the individual sensor responses indicated a wider contact footprint consistent with the observed lying posture.

The torso inclination angle represents one component of the posture assessment rather than an independent criterion for confirming a fall. The final fall decision is determined by combining visual posture and motion information with floor-sensor evidence and the temporal confirmation conditions defined in the decision-level fusion framework.

The fusion node entered the Fallen state upon confirming the following:

- A lying posture sustained beyond the minimum duration $T_{l}=2.0$ s;
- A standing-to-lying transition within the fall window $T_{f}=3.0$ s;
- A positive impact flag from the load sensor array.

### 3.2. YOLOv8 Skeletal Tracking in Fallen Posture

Visual inspection of the representative frame in Figure 5 indicates that YOLOv8n-pose maintained detectable upper-body skeletal keypoints in the lying posture, including the shoulders, elbows, and wrists. Similar qualitative behaviour was observed across the experimental trials; however, keypoint localisation accuracy was not independently evaluated against manually annotated skeletal ground truth.

### 3.3. Confusion Matrix

Table 11 presents the confusion matrix for the proposed fused system across all experimental trials.

The confusion matrix shows that the proposed system correctly detected 72 of 80 fall trials and correctly rejected 78 of 80 activities of daily living. The low number of false positives demonstrates the benefit of requiring complementary evidence from both skeletal pose estimation and floor-based force sensing before confirming a fall event.

### 3.4. Quantitative Performance Results

#### Per-Participant Detection Performance

Table 12 summarises fall detection performance for each participant individually. Each participant completed two repetitions of each of the five fall scenarios (10 fall trials per participant; 80 total).

Two participants (P2 and P6) each generated one false alarm, both from rapid intentional lying-down trials. These are discussed further in Section 3.7.

The overall sensitivity across all participants was 0.900 (72 of 80 fall trials detected). Participants P3 and P5—both males with higher body mass (85.1 kg and 92.3 kg, respectively)—achieved perfect detection sensitivity, generating impact scores consistently above threshold across all fall types. Participants P2 and P4 exhibited the lowest per-participant sensitivity (0.800) and also produced relatively low load-sensor impact responses during several slow-collapse trials. These observations suggest that low-impact fall dynamics may reduce the likelihood of satisfying the fixed impact threshold. Although both participants were among the lighter members of the cohort, the small sample size does not permit conclusions regarding an independent relationship between body mass and detection sensitivity. Similarly, participant-level differences in detection latency are reported descriptively and should not be interpreted as evidence of a mass-dependent effect.

### 3.5. Performance by Fall Type

Detection performance varied across the five simulated fall scenarios, as summarised in Table 13. Forward and stumble falls were detected with perfect sensitivity in the present dataset, whereas lateral and backward falls each achieved a sensitivity of 0.875. Slow-collapse events were the most challenging, with a sensitivity of 0.750. Lower detection performance was observed primarily during slow-collapse, lateral, and backward fall scenarios, whereas forward and stumble falls were detected with perfect sensitivity in the present dataset.

Overall, the proposed framework demonstrated encouraging performance across the evaluated simulated fall scenarios, indicating that the complementary sensing modalities effectively capture both rapid and gradual fall dynamics.

Forward falls and stumble falls achieved perfect sensitivity (1.000), producing consistent and pronounced feature responses across all participants: large ${\dot{y}}_{\mathrm{trunk}}$, rapid torso-inclination transition, and clearly supra-threshold impact scores. Lateral and backward falls yielded sensitivity of 0.875; the four missed detections (two lateral, two backward) occurred when participants fell partially outside the load-sensor array footprint, yielding load-sensor responses below the impact threshold and precluding fusion confirmation despite correct vision-based skeletal posture classification.

Slow-collapse falls produced the lowest sensitivity (0.750), with 12 of 16 trials correctly detected. The four missed slow-collapse events occurred in participants P2 and P4, with two false-negative trials for each participant. In these cases, the gradual descent produced trunk-centroid velocities below $\tau_{v}$ and load-sensor impact scores below $\tau_{\mathrm{impact}}$, preventing the temporal fusion criteria from being satisfied. Candidate-fall detection latency was also longest for slow-collapse events (2.21±0.55 s), reflecting their more gradual transition dynamics.

#### 3.5.1. False-Positive Performance Across ADL Scenarios

Table 14 presents false alarm results across the five ADL scenarios. Each ADL was performed twice by each of eight participants, yielding 16 trials per ADL type (80 ADL trials total).

The overall false-positive rate was 0.025 (two false alarms in 80 ADL trials). Both false alarms arose from the intentional lying-down scenario and were associated with rapid, forceful descents to the floor by participants P2 and P6, which transiently exceeded both $\tau_{v}$ and $\tau_{\mathrm{impact}}$. All other ADL scenarios produced zero false alarms in the present experimental dataset, suggesting that the minimum lying-duration filter $T_{l}$ and fall time window $T_{f}$ contributed to suppressing transient postural changes associated with bending, kneeling, sitting, and walking.

The proposed classifier generates a deterministic binary decision at a single predefined operating point rather than a continuous fall-probability score. ROC and precision–recall curves were therefore not constructed, as doing so would require systematic variation of the predefined decision thresholds. Such threshold sweeps were not performed on the main evaluation dataset because the thresholds had been fixed before testing. Similarly, false alarms per hour were not estimated because the experimental protocol consisted of discrete scripted trials rather than continuous monitoring over a defined exposure duration. Long-duration evaluation will be required to estimate false-alarm frequency under realistic monitoring conditions.

#### 3.5.2. Overall System Performance Metrics

Table 15 presents the aggregated performance metrics across all 160 experimental trials, comprising 80 simulated fall trials and 80 activities of daily living (ADLs).

The proposed fusion framework achieved a sensitivity of 0.900 (95% CI: 0.815–0.948), specificity of 0.975 (95% CI: 0.913–0.993), precision of 0.973 (95% CI: 0.907–0.993), accuracy of 0.938 (95% CI: 0.889–0.966), and an F1-score of 0.935.

The mean *candidate-fall* detection latency—defined as the elapsed time from ground-truth fall onset to the fusion node’s first identification of a fall transition event ($T_{\mathrm{candidate}}-T_{\mathrm{onset}}$)—was 1.80±0.41 s across true-positive detections. This is distinct from the final alarm latency, which additionally includes the mandatory $T_{l}=2.0$ s posture persistence window and is approximately 3.8 s on average. This two-stage design is deliberate: the candidate detection triggers the internal persistence timer, whilst the alarm is published only once sustained floor-level posture confirms a genuine fall. The distinction between candidate detection and final alarm generation is important when interpreting system responsiveness. The approximately 3.8 s final alarm delay reflects the deliberate use of the $T_{l}=2.0$ s persistence criterion to suppress transient false alarms; its suitability for a particular deployment context would depend on the corresponding care pathway and alarm-response requirements [17].

The high specificity indicates that the multimodal fusion strategy effectively limited false-positive detections. Fall confirmation required an appropriate temporal sequence of visual transition evidence, corroborating floor-sensor evidence, and sustained floor-level posture, thereby reducing false alarms from ADLs that produced postural transitions visually similar to falls.

#### 3.5.3. Participant-Level Variability Analysis

Inter-participant variability in detection performance provides insight into the influence of individual characteristics on system reliability. Table 16 summarises the mean feature values recorded at the frame of fall confirmation for true-positive detections, aggregated by participant.

Descriptive differences in the recorded features were observed across participants. In particular, participants with lower body mass tended to exhibit lower load-sensor impact scores, whereas several higher-body-mass participants produced larger impact responses. For example, the mean impact scores for P2, P4, and P8 were 0.41, 0.38, and 0.43, respectively, compared with 0.67, 0.71, and 0.61 for P3, P5, and P7. The lower impact responses observed for some participants approached the detection threshold $\tau_{\mathrm{impact}}=0.30$ and may have contributed to missed detections, particularly during slow-collapse events. However, these observations are descriptive only. Given the small sample size (n=8), the present study was not designed or statistically powered to establish body mass as an independent determinant of impact response or fall detection performance.

### 3.6. Sensor Fusion Contribution Analysis

To quantify the contribution of each sensing modality, two single-modality baseline configurations were evaluated using the same 160-trial dataset:

1. **Vision-Only**: A fall was declared from skeletal pose-derived postural and temporal evidence, without requiring load-sensor corroboration.
2. **Load-Only**: A fall was declared using impact score and spatial load-distribution evidence without pose-estimation information.

The vision-only and load-only baselines were not independently re-optimised. Each baseline used the corresponding branch-specific decision parameters established during the same pilot-stage threshold-selection process used for the fused framework. No parameters were adjusted using the 160-trial evaluation dataset (see Table 17).

The vision-only configuration achieved the highest sensitivity (0.938), as it did not require floor-sensor corroboration and was therefore less affected by incomplete load-sensor coverage. However, specificity decreased to 0.863, with 11 false-positive detections, indicating that skeletal posture and temporal evidence alone were less effective at distinguishing some fall-like ADLs from genuine falls. The load-only configuration achieved a sensitivity of 0.838 and specificity of 0.938. Its missed detections were associated primarily with low-impact slow-collapse events and falls occurring outside the effective instrumented region.

The fused configuration achieved an F1-score of 0.935, compared with 0.904 for the vision-only baseline and 0.882 for the load-only baseline. The number of false-positive detections decreased from 11 in the vision-only configuration to 2 in the fused configuration, corresponding to an 81.8% reduction. However, sensitivity decreased from 93.8% to 90.0%, representing a 3.8-percentage-point reduction in the proportion of falls correctly detected. This trade-off reflects the additional requirement for corroborating floor-sensor evidence, which can suppress false alarms but may also prevent fall confirmation when floor-sensor coverage is incomplete or impact responses are insufficient. Therefore, the present findings demonstrate improved specificity and F1-score under the evaluated conditions, rather than an improvement across all performance metrics.

### 3.7. Failure Mode Analysis

#### 3.7.1. False Negatives

Eight missed fall detections were identified and categorised across the cohort. Cross-referencing the per-participant detection table (Table 12) with each trial record confirms the following attribution:

**Falls outside Load-Sensor Array Boundary** (four cases): Participants P1 (1 FN), P6 (1 FN), P7 (1 FN), and P8 (1 FN) each had one lateral or backward fall trial in which the subject landed partially or wholly outside the instrumented floor zone. In these cases, the load-sensor array registered an impact score below $\tau_{\mathrm{impact}}$ and insufficient load to satisfy the lying-like load-distribution criterion. Fusion confirmation was therefore not achieved despite correct vision-based skeletal posture classification. This failure mode is attributable to the finite spatial coverage of the load-sensor array and is independent of participant body mass.

**Slow Collapse with Insufficient Impact** (four cases): Participants P2 (2 FN) and P4 (2 FN) each had two slow-collapse trials in which the gradual floor descent generated a trunk-centroid velocity below $\tau_{v}$ and a load-sensor impact score below $\tau_{\mathrm{impact}}$, leaving the fusion node in the Transitioning state beyond the fall window $T_{f}$. In all four cases, the vision-only classifier correctly identified a lying posture, confirming that the load-sensor impact criterion was the binding constraint. Both participants were among the lightest cohort members (58.4 kg and 61.7 kg respectively), and their mean impact scores (Table 16: 0.41 and 0.38) were closest to $\tau_{\mathrm{impact}}=0.30$, consistent with reduced impact force during controlled slow descents. Given the small sample size, these observations are descriptive and should not be interpreted as establishing body mass as an independent determinant of detection performance.

The attribution above is summarised in Table 18 and is fully consistent with the per-participant false-negative counts reported in Table 12: P1 = 1, P2 = 2, P4 = 2, P6 = 1, P7 = 1, P8 = 1 (total = 8).

#### 3.7.2. False Positives

Two false alarms were recorded, both during intentional lying-down trials:

- **P2 (Trial 2, intentional lying)**: Participant descended unusually rapidly, generating a transient normalised trunk-centroid velocity ${\dot{y}}_{\mathrm{trunk}}$ slightly above the threshold $\tau_{v}=0.18\;s^{-1}$, coincident with an impact score of 0.34, marginally above $\tau_{\mathrm{impact}}=0.30$. Post hoc telemetry inspection revealed that the spatial load-distribution pattern did not provide sufficiently strong evidence of sustained floor contact prior to alarm generation.
- **P6 (Trial 1, intentional lying)**: Similar mechanics to the P2 case; a forceful descent generated an impact score of 0.32. The participant’s movement speed was atypically high relative to the calibration dataset, placing the event in a region of parameter space not well represented during threshold determination.

Both false alarms share a common root cause: rapid intentional floor descent that falls within the parameter overlap region between fast ADLs and genuine falls. This overlap is irreducible to some degree by any threshold-based system. These observations suggest that incorporating an additional spatial load-distribution criterion may help reduce false alarms arising from rapid intentional floor descent. However, this modification requires prospective evaluation to determine its effects on both specificity and sensitivity.

### 3.8. Computational Performance

YOLOv8n-pose inference on the host GPU averaged 38.2±2.1 FPS across all experimental sessions, comfortably exceeding the 30 FPS camera acquisition rate and confirming that pose estimation introduced no processing bottleneck. The measured frame-processing latency averaged 47±8 ms. This computational latency should be distinguished from the event-level fall detection delay, which also includes the temporal confirmation requirements of the state machine. In particular, the final fall alarm is generated only after the required postural and floor-sensor evidence has satisfied the specified temporal conditions. Peak CPU utilisation across all ROS2 nodes reached 34% during concurrent inference and load sensor processing, indicating that the processing workload remained well within the capabilities of the host workstation, and motivating future evaluation on more resource-constrained embedded hardware.

Table 19 summarises per-node computational contributions.

The pose estimator node dominates CPU utilisation (19.4%), as expected given the convolutional inference workload. The fusion node contributes minimal overhead (2.9%), reflecting the computational simplicity of the state-machine logic relative to the perception tasks. The total pipeline latency of 47 ms is well within real-time constraints and substantially below the detection latency of 1.80 s. These measurements demonstrate real-time operation on the evaluated host workstation. Performance on resource-constrained embedded or edge hardware was not evaluated in the present study.

## 4. Discussion

The proposed framework adopts decision-level fusion so that the vision and floor-sensing branches can be processed independently without requiring sample-level alignment of their heterogeneous measurements. This modular design is advantageous for the present implementation. However, feature-level and signal-level fusion architectures were not implemented as comparative baselines; consequently, the present results do not establish superiority of decision-level fusion over alternative fusion strategies. Likewise, although the vision-only baseline retains temporal postural reasoning, a more extensive ablation would be required to fully separate the effects of multimodality, fusion level, and temporal filtering.

### 4.1. Interpretation of Results

The experimental evaluation demonstrates that the proposed multimodal framework improves fall detection by combining complementary skeletal postural and motion information from the vision subsystem with physical floor-interaction evidence from the load-sensing platform. Vision-based skeletal tracking captures changes in body orientation and normalised trunk-centroid motion, whereas the distributed load-sensing platform provides information regarding impact and spatial load distribution during human movement. The combination of these complementary sensing modalities enables reliable discrimination between genuine falls and activities of daily living that often produce similar visual appearances.

The proposed framework employs decision-level fusion to enable independent processing of the vision and floor-sensing branches without requiring sample-level alignment of the heterogeneous sensor measurements. This modularity is advantageous for the present implementation. However, feature-level and signal-level fusion architectures were not evaluated as comparative baselines, and the present results therefore do not establish that decision-level fusion is superior to alternative fusion strategies.

The temporal state machine further contributes to the observed performance by incorporating temporal consistency into the decision process. Instead of relying on instantaneous posture estimates, fall decisions are confirmed only when the required sequence of visual and floor-sensor evidence satisfies the temporal transition criteria. Consequently, rapid activities such as sitting, kneeling, bending, or intentional lying are less likely to generate false alarms, explaining the substantial reduction in false-positive detections observed during experimental evaluation.

### 4.2. Comparison with Existing Work

Existing fall detection systems generally rely on wearable inertial sensors, vision-based monitoring, or ambient sensing technologies. Wearable systems typically achieve high detection sensitivity but require continuous user compliance and regular maintenance, limiting long-term deployment among older adults. Vision-based approaches provide richer contextual information but remain susceptible to lighting variations, occlusions, and difficulties distinguishing falls from intentional floor activities. Ambient sensing methods preserve user privacy but often lack sufficient contextual information to distinguish accidental falls from normal daily activities.

The proposed framework addresses these limitations by integrating complementary information from both vision- and floor-based sensing. Unlike conventional vision-only approaches, the additional load-sensor-derived floor-load measurements provide complementary evidence of floor interaction and impact-related changes, substantially reducing false-positive detections. Compared with wearable solutions, the proposed system operates entirely without body-mounted devices, improving user acceptance and reducing dependence on user compliance.

Although the obtained performance is encouraging, direct quantitative comparison with published fall detection systems should be interpreted cautiously because of differences in participant populations, sensing modalities, activity protocols, dataset sizes, and evaluation procedures. The temporal persistence requirement introduces an explicit trade-off between false-alarm suppression and alarm delay. Requiring the lying condition to persist for $T_{l}=2.0$ s reduces the likelihood that brief floor-level postures or transient classification errors generate an alarm, but this necessarily delays final alarm confirmation. In applications requiring rapid escalation following a fall, this delay must be considered alongside the reduction in false-positive events. The present study does not define the measured alarm delay as clinically acceptable, since the appropriate response-time requirement depends on the deployment context, care pathway, and alarm-management protocol. Future work will investigate adaptive persistence intervals and multi-stage alerts in which an early candidate-fall warning can precede final confirmation.

### 4.3. Practical Implications

The proposed multimodal architecture has potential for future deployment in residential homes, assisted-living facilities, rehabilitation centres, and hospital environments where continuous, unobtrusive monitoring is required. Because the system does not rely on wearable devices, it avoids common problems associated with user compliance, charging requirements, and device removal. Furthermore, the distributed sensing architecture enables continuous monitoring without requiring users to modify their daily routines.

The modular design of the framework also facilitates future integration with smart-home technologies and healthcare monitoring systems. Additional sensing modalities, including radar, thermal imaging, inertial sensing, or environmental sensors, could be incorporated within the existing decision-level fusion framework with minimal modification to the current system architecture. Consequently, the proposed framework provides a flexible platform for future intelligent ambient assisted-living applications.

Slow-collapse events represent a specific weakness of the present implementation. Only 12 of 16 slow-collapse trials were detected, corresponding to a sensitivity of 75%. Gradual descent can produce weaker trunk-motion and floor-impact signatures than rapid falls, reducing the probability that the fixed velocity and impact criteria are satisfied. Future development should therefore consider additional temporal and load-distribution evidence that does not depend primarily on a pronounced impact event.

### 4.4. Limitations and Future Work

Several limitations should be considered when interpreting the present findings. First, the evaluation involved eight healthy adults aged 19–34 years. Although each participant completed repeated fall and ADL trials, yielding 160 recorded trials, these observations should not be interpreted as 160 independent participant-level samples. The independent human sample comprised eight participants, with repeated observations obtained from each individual. Consequently, the reported trial-level performance characterises system behaviour within this controlled feasibility evaluation and should not be interpreted as population-level evidence of performance in older adults or people with mobility impairment.

This distinction is particularly important because fall dynamics, protective responses, transition speed, impact magnitude, and post-fall movement in young healthy volunteers performing instructed falls may differ from genuine accidental falls experienced by older or mobility-impaired individuals. Recruitment of vulnerable populations for deliberate fall simulation also presents ethical and safety constraints. The present study should therefore be regarded as an initial controlled technical validation. Future work requires a larger and more diverse cohort, including older adults and participants with mobility-related characteristics where ethically and clinically appropriate, followed by prospective evaluation of naturally occurring events in representative living environments.

Second, the experimental evaluation was performed within a controlled indoor environment. Although the test environment incorporated variations in lighting and partial occlusions, further validation in occupied residential and assisted-living environments is required to assess long-term robustness under unconstrained conditions.

Third, the current implementation relies on empirically selected decision thresholds established using preliminary pilot trials involving the same eight participants as the main evaluation. Although the pilot trials were excluded from the main evaluation dataset and the selected thresholds were fixed before the evaluation, the participant populations were not independent. Consequently, the present results do not establish subject-independent generalisation, and participant-specific movement characteristics may have influenced the observed performance. Future work should investigate participant-independent evaluation using leave-one-subject-out or other subject-wise validation protocols, with threshold selection performed exclusively using the corresponding training participants. Evaluation using an independent cohort is also required to assess generalisation to previously unseen individuals.

Fourth, the present study also does not include leave-one-subject-out evaluation, a systematic comparison of alternative fusion strategies, or inferential statistical testing of performance differences. The reported results therefore describe observed performance under the implemented decision-level fusion strategy and the experimental protocol used, rather than establishing superiority across alternative fusion architectures or statistically significant improvements beyond the evaluated sample. Future work should address these questions using participant-wise validation, controlled comparisons between fusion approaches, and statistical methods that account for repeated observations within participants.

Fifth, the present evaluation was restricted to single-participant scenarios. The behaviour of the proposed fusion framework in multi-occupant environments has not yet been established, particularly where visual skeletal observations must be associated with spatially overlapping floor-sensor responses. Future work should therefore investigate multi-person tracking and sensor-to-person association strategies.

Sixth, the finite spatial coverage of the floor-mounted load-sensor array affected detection performance. Four false-negative detections occurred when participants landed partially outside the instrumented region, reducing the availability of floor-sensor corroborating evidence. Larger or modular sensing surfaces, together with fusion strategies capable of maintaining reliable operation when floor-sensor coverage is incomplete, should therefore be investigated.

Beyond addressing these limitations, future work will investigate adaptive threshold optimisation and machine-learning-based decision fusion to improve generalisation across different users and environments. Additional sensing modalities, including millimetre-wave radar, thermal imaging, and wireless sensing technologies, could also be explored to improve robustness under challenging environmental conditions.

Larger-scale validation involving older adults and individuals with mobility impairments will be required to establish the clinical and real-world applicability of the proposed framework. Longer-term deployment in residential and assisted-living environments should also examine system reliability, user acceptability, privacy considerations, and integration with remote healthcare platforms for timely intervention following detected fall events. Finally, activities were performed in a predefined rather than randomised sequence. Although thresholds were fixed prior to the main evaluation, a fixed activity order may introduce familiarisation or order effects. Future evaluations should therefore randomise or counterbalance the activity order. The controlled protocol also does not reproduce the full variability encountered during continuous residential monitoring, including unscripted activity patterns, changing environmental configurations, long-duration operation, and interactions occurring outside the instrumented region. Accordingly, the present results should not be interpreted as evidence of real-world residential performance. Such performance requires prospective evaluation under unconstrained and long-duration deployment conditions. The current floor-sensing platform was developed as an experimental prototype and should not be interpreted as a deployment-ready residential flooring solution. Practical installation in occupied homes would require more unobtrusive integration, such as flush-mounted or modular sensing tiles beneath conventional flooring. The present study did not evaluate whether awareness of the instrumented surface influences user behaviour, gait, or acceptability. Installation complexity, long-term durability, maintenance, and user perception should therefore be evaluated in future residential studies.

## 5. Conclusions

This paper presents a real-time, non-wearable multimodal fall detection framework integrating YOLOv8-based skeletal pose estimation with a distributed floor-mounted load-sensing platform through decision-level fusion. Evaluation across 160 controlled trials involving eight healthy participants demonstrated the technical feasibility of combining skeletal posture and motion information with floor-load evidence under the conditions investigated. The system achieved an F1-score of 0.935, sensitivity of 90.0%, and specificity of 97.5% on the evaluated dataset. These findings support the potential value of complementary sensing, but they do not establish subject-independent generalisation of performance in older adults and other populations at elevated risk of falling. Further participant-independent and real-world validation is required. Furthermore, computational evaluation confirmed that the complete framework operates in real time, supporting the technical feasibility of real-time continuous monitoring.

Although the current study involved healthy adult participants and was conducted under controlled indoor conditions, the results demonstrate the technical feasibility of the proposed approach within the evaluated setting. The modular architecture provides a foundation for further development, including the integration of additional sensing modalities and future evaluation in residential homes, rehabilitation facilities, and healthcare environments.

Future work will focus on large-scale clinical validation involving older adults and individuals at increased risk of falling, evaluation in real-world residential environments, and the investigation of adaptive decision thresholds and learning-based sensor fusion strategies to further improve robustness and generalisation across diverse users and environmental conditions.

## Figures and Tables

**Table 1:** Conceptual comparison of representative fall detection sensing approaches and the proposed framework.

Approach	Primary Sensor(s)	Pose Estimation	Sensor Fusion	Real-Time	Main Limitations
Wearable IMU systems [3]	Accelerometer/IMU	No	No	Yes	Require continuous user compliance, charging, and correct device placement.
Vision-based methods [4]	RGB camera	Limited	No	Moderate	Sensitive to lighting variation, occlusion, camera placement, and privacy concerns.
Ambient sensing approaches [5]	Floor vibration/acoustic/pressure sensors	No	No	Yes	Limited contextual information and difficulty distinguishing falls from other impact events.
YOLOv8 pose estimation [9]	RGB/RGB-D camera	Yes	No	Yes	Provides accurate posture estimation but cannot directly measure impact forces.
General multisensor fusion approaches [10,11]	Multiple sensors	Dependent	Feature/decision level	Dependent	Often require careful synchronisation, calibration, and application-specific optimisation.
Proposed framework	RGB-D camera + load-sensing array	YOLOv8 skeletal pose	Decision-level fusion	Yes	Limited by floor sensor coverage, but improves robustness through complementary skeletal-pose and floor-based force sensing.

**Table 2:** Representative recent fall detection approaches and comparison with the proposed framework. Reported values are not directly comparable, because datasets and protocols differ between studies.

Study	Sensing Modality	Method	Evaluation	Key Distinction
Bui et al. (2024) [12]	RGB + WiFi CSI	YOLOv5s + CNN–LSTM	Study-specific dataset	Cross-modal vision/RF sensing
Zheng et al. (2024) [13]	RGB	Improved YOLOv8	Study-specific dataset	Deep learning vision detector
Khawam et al. (2025) [14]	RGB	YOLOv8-Pose	186 videos	Pose-based vision detection
Proposed framework	RGB + distributed load sensing	YOLOv8n-pose + temporal decision fusion	160 trials, 8 participants	Complementary skeletal and floor-interaction evidence

**Table 3:** Summary of the proposed multimodal fall detection pipeline.

Stage	Input	Output
Image acquisition	RGB stream (depth available)	RGB image frames
YOLOv8 pose estimation	RGB image	17 skeletal keypoints
Vision feature extraction	Keypoints	Torso angle, trunk centroid, velocity
Load-sensing acquisition	Digitised load-cell outputs	Load measurements
Load feature extraction	Load-sensing measurements	Load centroid, total load, impact score
Decision-level fusion	Vision + load decisions	Fall/ADL classification

**Table 4:** Hardware components used in the proposed multimodal fall detection framework.

Component	Model	Purpose
RGB-D Camera	Intel RealSense D435	RGB-D image acquisition
Pose Estimation	YOLOv8n-pose	Human pose estimation
Force Sensors	Load cells and Hx-711 ADC	Floor-load and impact sensing
Microcontroller	Arduino Mega 2560	Data acquisition
Processing Unit	Desktop workstation with NVIDIA RTX GPU	Real-time processing
Software Framework	ROS2	Communication and integration

**Table 5:** Intel RealSense D435 camera configuration and data usage.

Parameter	Value
Camera model	Intel RealSense D435
RGB resolution	640×480 pixels
RGB frame rate	30 FPS
Available depth technology	Active stereo
Stream used for pose estimation	RGB
Pose representation	2D skeletal keypoints
Depth used in fall classification	No
Mounting height	1.9 m
Camera orientation	Downward-facing

**Table 6:** Decision thresholds and system parameters used by the proposed fall detection framework.

Parameter	Symbol	Value	Purpose
Keypoint confidence	$s_{j}$	0.50	Reject unreliable skeletal keypoints
Upright posture	$\tau_{\alpha ,1}$	$30^{\circ }$	Upper limit for upright posture
Lying posture	$\tau_{\alpha ,2}$	$60^{\circ }$	Identify near-horizontal posture
Trunk-centroid velocity	$\tau_{v}$	$0.18\;s^{-1}$	Detect rapid downward image-plane motion
Impact	$\tau_{\mathrm{impact}}$	0.30	Detect significant floor impact
Minimum lying duration	$T_{l}$	2.0 s	Confirm sustained lying posture
Fall transition window	$T_{f}$	3.0 s	Maximum fall-to-lying duration
Ground-truth association window	$T_{g}$	5.0 s	Associate a generated alarm with the corresponding annotated fall event
Camera frame rate	$f_{c}$	30 Hz	RGB image acquisition frequency
Load-sensor sampling rate	$f_{s}$	50 Hz	Force-sensor sampling frequency

**Table 7:** Vision-based skeletal descriptors extracted from the estimated human pose.

Feature	Symbol	Purpose
Torso inclination	α	Body orientation
Normalised trunk centroid	$y_{\mathrm{trunk}}$	Vertical trunk position
Trunk-centroid velocity	${\dot{y}}_{\mathrm{trunk}}$	Rapid downward motion
Keypoint confidence	$s_{j}$	Measurement reliability

**Table 8:** Load-based features extracted from the distributed Load sensing platform.

Feature	Symbol	Purpose
Total Load	$L_{\mathrm{total}}$	Overall applied load
Load Centroid	$C_{L}$	Spatial load distribution
Impact Score	*I*	Rapid impact detection

**Table 9:** Participant characteristics.

ID	Sex	Age	Mass (kg)	Height (cm)
P1	M	24	78.2	181
P2	F	22	58.4	165
P3	M	31	85.1	178
P4	F	19	61.7	162
P5	M	28	92.3	185
P6	F	26	64.0	169
P7	M	34	80.5	176
P8	F	23	59.8	164
**Mean**	–	**25.9**	**72.5**	**172.5**

**Table 10:** ROS2 node graph: nodes, topics, and update rates.

Node	Publishes	Rate	Purpose
realsense_node	/camera/color/image_raw	30 Hz	RGB-D acquisition
pose_estimator_node	/pose_features	30 Hz	YOLOv8 inference and skeletal feature extraction
load_sensor_node	/load_features	50 Hz	Arduino serial parsing and load feature computation
fusion_node	/fall_alarm, /system_state	Event-driven	Temporal state machine and fall alarm generation

**Table 11:** Confusion matrix for the proposed multimodal fall detection system.

	Predicted Fall	Predicted Non-Fall
**Actual Fall**	72	8
**Actual Non-Fall**	2	78

**Table 12:** Per-participant fall detection performance (10 fall trials each).

Participant	Mass (kg)	TP	FN	Sensitivity	FP	Candidate Latency ^†^ (s)
P1	78.2	9	1	0.900	0	1.71±0.31
P2	58.4	8	2	0.800	1	2.03±0.58
P3	85.1	10	0	1.000	0	1.52±0.28
P4	61.7	8	2	0.800	0	2.14±0.63
P5	92.3	10	0	1.000	0	1.48±0.22
P6	64.0	9	1	0.900	1	1.89±0.41
P7	80.5	9	1	0.900	0	1.67±0.35
P8	59.8	9	1	0.900	0	1.97±0.49
**Total**	–	**72**	**8**	**0.900**	**2**	1.80±0.41

^†^ Latency values represent candidate-fall detection latency ($T_{\mathrm{candidate}}-T_{\mathrm{onset}}$), not the final alarm latency. The final alarm is issued after an additional $T_{l}=2.0$ s posture persistence window.

**Table 13:** Fall detection results by fall type (8 participants × 2 repetitions = 16 trials per type).

Fall Type	Trials	TP	FN	Sensitivity	Mean Candidate Latency (s)
Forward fall	16	16	0	1.000	1.51±0.24
Lateral fall	16	14	2	0.875	1.78±0.39
Backward fall	16	14	2	0.875	1.83±0.44
Slow collapse	16	12	4	0.750	2.21±0.55
Stumble fall	16	16	0	1.000	1.63±0.31
**Overall**	**80**	**72**	**8**	**0.900**	1.80±0.41

**Table 14:** False-positive results across ADL scenarios (8 participants × 2 repetitions = 16 trials per type).

ADL Scenario	Trials	TN	FP	FPR
Intentional lying down	16	14	2	0.125
Rapid sitting to floor	16	16	0	0.000
Bending to pick up object	16	16	0	0.000
Kneeling and standing	16	16	0	0.000
Walking through/exiting	16	16	0	0.000
**Overall**	**80**	**78**	**2**	**0.025**

**Table 15:** Overall system performance metrics across 160 experimental trials.

Metric	Value
Total trials	160
Fall trials	80
ADL trials	80
True positives (TP)	72
False negatives (FN)	8
True negatives (TN)	78
False positives (FP)	2
Sensitivity (recall)	0.900 (95% CI: 0.815–0.948)
Specificity	0.975 (95% CI: 0.913–0.993)
Precision	0.973 (95% CI: 0.907–0.993)
Accuracy	0.938 (95% CI: 0.889–0.966)
F1-score	0.935
Mean candidate-fall detection latency	1.80±0.41s
Mean final alarm latency (approx.)	≈3.8s

**Table 16:** Mean feature values at fall confirmation across participants (true positives only).

ID	Tilt α (°)	$y_{\mathrm{trunk}}$	${\dot{y}}_{\mathrm{trunk}}$	Relative Total Load (a.u.)	Impact Score
P1	71.3±8.2	0.731±0.041	0.198±0.031	76.8±5.1	0.58±0.09
P2	68.9±9.7	0.718±0.053	0.171±0.044	55.2±6.3	0.41±0.12
P3	74.1±6.4	0.748±0.038	0.214±0.028	83.7±4.8	0.67±0.08
P4	67.4±10.1	0.712±0.061	0.163±0.049	58.9±7.2	0.38±0.14
P5	76.2±5.9	0.761±0.034	0.228±0.025	90.1±5.5	0.71±0.07
P6	69.8±8.8	0.724±0.047	0.183±0.038	61.4±6.0	0.44±0.11
P7	72.5±7.3	0.739±0.043	0.204±0.033	78.3±4.9	0.61±0.09
P8	69.1±9.2	0.720±0.055	0.176±0.042	57.6±6.8	0.43±0.13

**Table 17:** Comparison of the fused system with the single-modality baselines across the same 160-trial dataset.

Configuration	Sensitivity	Specificity	Precision	F1	FP Count
Vision-only	0.938	0.863	0.872	0.904	11
Load-only	0.838	0.938	0.931	0.882	5
**Fused (proposed)**	**0.900**	**0.975**	**0.973**	**0.935**	**2**

**Table 18:** False-negative attribution by participant and cause.

Participant	FN (Total)	Load Sensor Boundary	Slow Collapse
P1	1	1	0
P2	2	0	2
P4	2	0	2
P6	1	1	0
P7	1	1	0
P8	1	1	0
**Total**	**8**	**4**	**4**

**Table 19:** Computational performance by ROS2 node.

Node	Rate (Hz)	Mean CPU (%)	Mean Latency (ms)
realsense_node	30	8.1	5
pose_estimator_node	30	19.4	26
load_sensor_node	50	3.2	6
fusion_node	30	2.9	10
**Total pipeline**	–	**33.6**	**47**

## Data Availability

The data supporting the findings of this study are available from the corresponding author upon reasonable request, subject to applicable ethical and data-protection requirements.
